# SpliceGrapher: detecting patterns of alternative splicing from RNA-Seq data in the context of gene models and EST data

**DOI:** 10.1186/gb-2012-13-1-r4

**Published:** 2012-01-31

**Authors:** Mark F Rogers, Julie Thomas, Anireddy SN Reddy, Asa Ben-Hur

**Affiliations:** 1 Department of Computer Science, 1873 Campus Delivery, Colorado State University, Fort Collins, CO 80523-1873, USA; 2 Department of Biology and Program in Molecular Plant Biology, 1878 Campus Delivery, Colorado State University, Fort Collins, CO 80523-1878, USA; 3 Department of Statistics, 1877 Campus Delivery, Colorado State University, Fort Collins, CO 80523-1877, USA

## Abstract

We propose a method for predicting splice graphs that enhances curated gene models using evidence from RNA-Seq and EST alignments. Results obtained using RNA-Seq experiments in *Arabidopsis thaliana *show that predictions made by our SpliceGrapher method are more consistent with current gene models than predictions made by TAU and Cufflinks. Furthermore, analysis of plant and human data indicates that the machine learning approach used by SpliceGrapher is useful for discriminating between real and spurious splice sites, and can improve the reliability of detection of alternative splicing. SpliceGrapher is available for download at http://SpliceGrapher.sf.net.

## Background

Deep transcriptome sequencing (RNA-Seq) with next-generation sequencing (NGS) technologies is providing unprecedented opportunities for researchers to probe the transcriptomes of many species [1-5]. An important goal in these studies is to assess the extent of alternative splicing (AS), a process that increases transcriptome and proteome diversity, and plays a key role in regulating gene expression and protein function [6,7]. Although it is inexpensive and easy to obtain whole transcriptome data using RNA-Seq, one limitation has been the lack of versatile methods to analyze these data. Consequently, there is an increasing demand for methods that can use the short reads produced in these studies to predict patterns of AS.

The sequences produced by NGS methods have characteristics that complicate the task of identifying the mRNA transcripts represented in a sample. A sequencing read may consist of fewer than 40 nucleotides, making it difficult to identify a unique origin within a reference sequence. In addition, NGS base-call error rates tend to increase with read length, raising the chance of a mismatch when aligning a read to a reference sequence [8]. These ambiguities are exacerbated by the presence of paralogous genes that can give rise to reads that align well in multiple locations. Much of the work on analyzing NGS reads has focused on aligning reads within exonic regions, and many methods exist for the problem of aligning reads without gaps-for example, MAQ [9], PASS [10] and BowTie [11].

Reads that span splice junctions introduce additional challenges. A splice junction may occur anywhere within a short read, so the read may have just a few bases on one side of a junction. Such a short sequence may align in multiple locations, making it difficult to identify its true origin. One can use heuristics to restrict the number of candidate locations: for example, by establishing limits for permissible intron lengths, or by focusing on locations that are bounded by canonical GT-AG or GC-AG splice-site dimers. Several spliced alignment algorithms exist that use these and other approaches to identify unique alignments for spliced reads [12-17].

The first studies that used RNA-Seq data to predict AS focused on exon-skipping events, the most prevalent form of AS in mammals (see, for example, [1,18-21]). To identify splice junctions recapitulated in short read data, these studies used exon sequences flanking annotated splice sites to produce a database of splice junction sequences. Using novel combinations of known acceptor and donor sites, researchers can create a database that consists of both known and putative splice junction sequences. RNA-Seq reads that align to these putative sequences then provide evidence for novel splicing events.

In this work we compare our approach with two methods: Cufflinks [22], and TAU [3]. Both methods were originally designed for *de novo *splice form prediction, but can make limited use of existing annotations. TAU predicts splice forms by assembling for each gene all feasible combinations of exons that have been identified in the alignment and spliced-alignment step. This approach ensures that no splice form will be overlooked, but it can produce a large number of transcripts that make it difficult to identify the most realistic predictions. Cufflinks uses a more sophisticated approach that seeks to identify the smallest set of mRNA transcripts that explain the observed data [22]. The Scripture method constructs a transcript graph on the basis of regions that exhibit statistically significant read depth compared to genomic background and predicts splice forms by considering all paths through the graph, an approach similar to TAU's [23]. Methods that perform *de novo *transcriptome assembly without requiring a reference genome-for example, Trinity [24] and ABySS [25]-are not included in our comparison.

**Figure 1:**
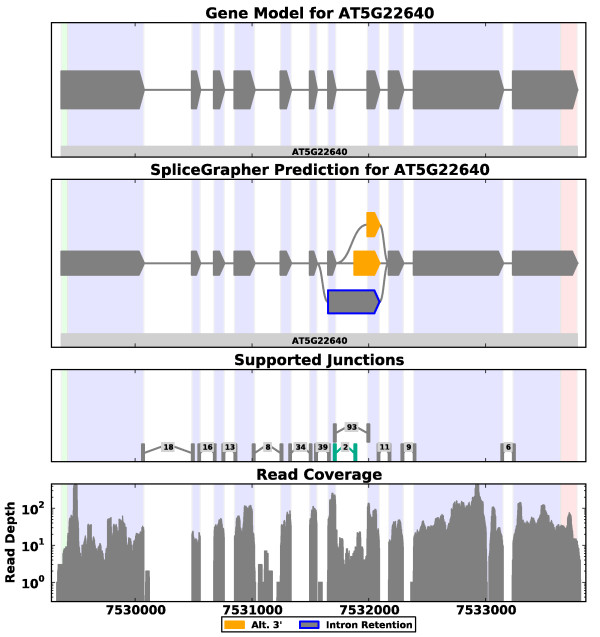
**Example of a predicted splice graph in *A. thaliana***. RNA-Seq alignment data were loaded along with gene model annotations to create a composite model that incorporates all available evidence. SpliceGrapher's visualization modules produce color-coded graphs based on the color scheme used by Sircah [29] that makes it easy to see exons and introns involved in AS events. RNA-Seq read coverage across one of the introns was sufficient to allow SpliceGrapher to identify an intron retention event (exon outlined in blue). In addition, a novel splice junction (highlighted in green) provided SpliceGrapher with evidence for an alternative 3' splicing event (highlighted in orange). The numbers associated with splice junctions indicate the number of reads that align across it. Vertical bands in the background depict exon boundaries in the original gene model.

To address ambiguities that inevitably arise when using short reads, and to take advantage of a rich and fast-evolving body of transcriptome data, we developed SpliceGrapher, a Python-based scripting tool designed to leverage gene annotations and ESTs, in addition to NGS reads. SpliceGrapher predicts 'splice graphs' (Figure 1), which capture in a single structure all the ways in which exons for a given gene may be assembled [26-32]. In these graphs, exons are depicted as nodes and introns are the edges that connect them. The compact structure allows researchers to visualize AS easily; and furthermore, we argue that NGS data rarely support the prediction of novel splice forms unambiguously. The splice graph structure permits SpliceGrapher to evaluate NGS data in the context of existing annotations, facilitates automated statistical analysis of AS events [31], and aids in comparing AS behaviour between gene families [32].

A few tools, such as Sircah, can produce splice graphs from conventional EST or cDNA alignments [29]. In previous work we enhanced Sircah's AS detection rules and extended the package to provide statistics and protein predictions for genome-wide studies of AS based on EST data [31]. With SpliceGrapher we extend this idea by incorporating multiple forms of data, including RNA-Seq reads, into splice graph predictions.

SpliceGrapher was designed from the outset to integrate RNA-Seq data, annotated gene models and EST alignments to produce comprehensive splice graph predictions. It applies inference rules to generate predictions that are compatible with all available evidence. The package includes flexible visualization tools that can depict splice graphs along with the evidence used to predict them. We compare SpliceGrapher's predictions with splice graphs generated from TAU and Cufflinks output based on RNA-Seq data and find that our results are more consistent with existing evidence from curated gene models.

## Results and discussion

### Splice graph prediction pipeline

**Figure 2:**
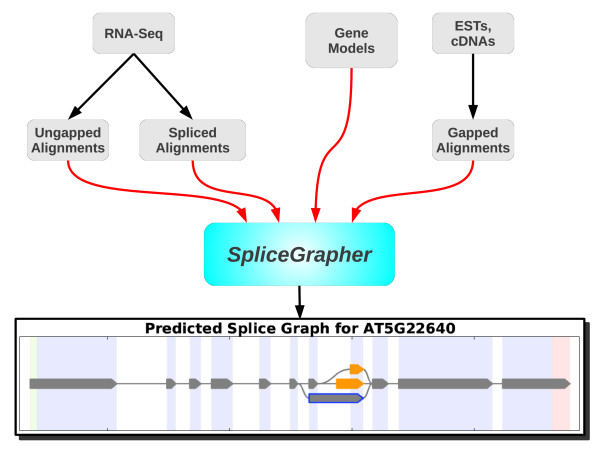
**Splice graph prediction pipeline**. SpliceGrapher predicts splice graphs using information from gene models, EST alignments and RNA-Seq data. RNA-Seq exonic alignments may be performed using any popular short-read alignment tool. RNA-Seq spliced alignments may be performed using a conventional short-read mapping tool with a database of splice junctions predicted by SpliceGrapher, or they may be performed using short-read spliced-alignment programs such as TopHat, followed by filtering using SpliceGrapher's database of predicted splice sites. SpliceGrapher incorporates all of this information to produce a comprehensive splice graph prediction.

SpliceGrapher is designed to enhance existing gene models using RNA-Seq and EST data. Relying on existing annotations as a baseline provides SpliceGrapher with a context in which to interpret short-read data. SpliceGrapher's splice graph prediction pipeline consists of the following steps (Figure 2): ungapped alignment of short reads to the reference genome, spliced alignment of reads that did not align in the first step, initial splice graph construction from the annotated gene models, assembly of exons from the ungapped short-read alignments, and insertion of the new exons into the splice graph using spliced alignments. SpliceGrapher accepts as input EST alignments as well; these are interpreted as splice graphs that SpliceGrapher merges with its gene model baseline graphs.

Figure 1 provides an example of a splice graph that SpliceGrapher predicted for a gene in *Arabidopsis thaliana*, along with the graph constructed from the gene model, the splice junctions that were recapitulated in the RNA-Seq data, and the read depth along the genomic region. SpliceGrapher combined these different forms of evidence and predicted only those novel splicing events it could resolve unambiguously.

**Figure 3:**
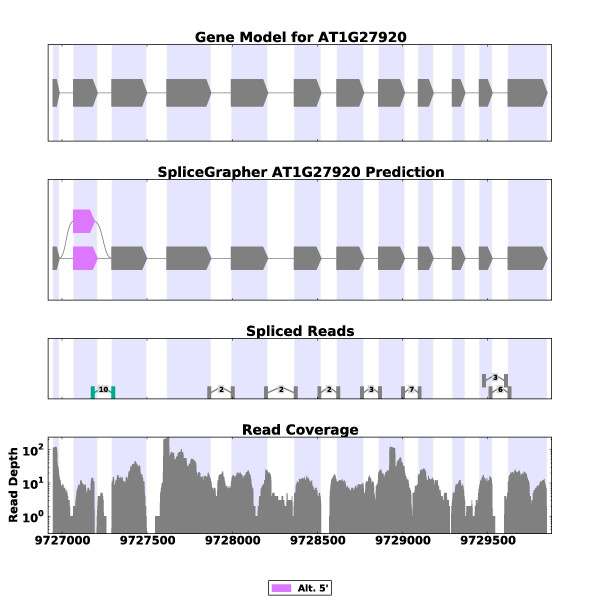
**Ambiguities in RNA-Seq data**. This figure demonstrates ambiguities that arise in RNA-Seq data that make isoform prediction challenging. Because there is read coverage across several introns, SpliceGrapher is not able to determine whether this is a result of a single intron retention event, or several independent events.

We first present results on RNA-Seq data from two plant genomes; applicability to mammalian genomes is demonstrated in a later section. We ran SpliceGrapher on short-read data from *A. thaliana *[3] and *Vitis vinifera *[33]. In *Arabidopsis *we provided SpliceGrapher with The *Arabidopsis *Information Resource (TAIR) version 9 of the genome annotations. New splice forms annotated in TAIR version 10 were reserved for assessing the ability of SpliceGrapher to detect novel annotations. SpliceGrapher predicted nearly 1,500 AS events that were not present in the TAIR9 version of the *A. thaliana *genome annotations. In *V. vinifera*, whose gene models showed no AS, SpliceGrapher predicted more than 2,600 events. The breakdown of those events by type (intron retention, exon skipping, and alternative 5' or 3') is found in Table 1. In plants, intron retention (IR) is the most prevalent form of AS [34,35] and accounts for more than 30% of AS events in the *A. thaliana *gene models (see also [35]). The novel exon-skipping (ES) and alternative 5' events predicted in *A. thaliana *are in proportions similar to those found in the gene models (Table 1), while alternative 3' events are higher. Notably, just 21% of the novel events are IR, compared to 33% in the gene models. IR events are more difficult to predict from RNA-Seq data because prediction depends on having read coverage across an intron's full length (Figure 3 and Materials and methods). We have also run SpliceGrapher on a dataset composed of longer 76-nucleotide reads in *A. thaliana*; despite having less reads (41 million compared to 284 million), SpliceGrapher was able to find close to 2,400 novel AS events in the new dataset, compared to close to 1,500 in the 32-nucleotide read data. Complete statistics for the new data are provided in Table S1 in Additional file 1. In *V. vinifera *the rate of IR prediction is even lower, at 13%. This can be explained by the longer introns in *V. vinifera*, where the average intron length we found in the annotated genes is 969 nucleotides (compared with 170 nucleotides for *A. thaliana*). The intron length influences the predicted IR rate for *V. vinifera *by making prediction of IR from RNA-Seq data more difficult. Furthermore, *V. vinifera *may have a lower IR rate to begin with, since low IR rates are observed in species with long introns [36].

**Table 1:** Alternative splicing events

	AS	AS events				
		
	genes	IR	ES	Alt. 5'	Alt. 3'	Total
***A. thaliana *models**	4,029	1,987 (33%)	550 (9%)	1,256 (21%)	2,145 (36%)	5,938
**SpliceGrapher**						
No ESTs	4,901	2,248 (30%)	714 (10%)	1,560 (21%)	2,866 (39%)	7,388
Novel	885	308 (21%)	164 (11%)	304 (20%)	721 (48%)	1,497
With ESTs	6,162	3,658 (33%)	994 (9%)	2,335 (21%)	4,128 (37%)	9,916
Novel	2,154	1,779 (34%)	444 (8%)	1,079 (20%)	1,983 (38%)	5,285
**Cufflinks**						
No gene models	1,263	449 (32%)	383 (28%)	237 (17%)	319 (23%)	1,388
Novel	699	429 (32%)	380 (28%)	232 (17%)	304 (23%)	1,345
With gene models	6,056	4,029 (39%)	2,857 (27%)	1,427 (14%)	2,106 (20%)	10,419
Novel	2,319	2,232 (38%)	2,550 (43%)	552 (9%)	594 (10%)	5,928
**TAU**						
No gene models	2,777	893 (17%)	475 (9%)	1,481 (27%)	2,555 (47%)	5,404
Novel	1,591	811 (16%)	460 (9%)	1,431 (28%)	2,351 (47%)	5,053
With gene models	10,458	94,571 (85%)	598 (1%)	5,972 (5%)	9,820 (9%)	110,961
Novel	8,364	94,124 (86%)	476 (0%)	5,697 (5%)	9,219 (8%)	109,516
***V. vinifera *models**	0	0 (0%)	0 (0%)	0 (0%)	0 (0%)	0
**SpliceGrapher**	2,039	347 (13%)	830 (31%)	640 (24%)	838 (32%)	2,655
**TAU**						
No gene models	3,099	531 (10%)	684 (13%)	1,321 (25%)	2,743 (52%)	5,279
With gene models	15,874	135,585 (72%)	4,938 (3%)	23,615 (13%)	24,406 (13%)	188,544
**Cufflinks**						
No gene models	1,057	324 (24%)	519 (39%)	140 (11%)	349 (26%)	1,332
With gene models	4,263	4,120 (34%)	3,148 (26%)	2,165 (18%)	2,818 (23%)	12,251

The number of AS events detected by SpliceGrapher, Cufflinks, and TAU compared with events inferred from the TAIR9 annotations. We track the following AS event types: intron retention (IR), exon skipping (ES), alternative 5' sites (Alt. 5') and alternative 3' sites (Alt. 3'). SpliceGrapher uses the TAIR9 gene models as a baseline, so it includes all of the same AS events along with additional events inferred from RNA-Seq data. Without gene models, nearly all TAU and Cufflinks predictions are novel AS events. With gene models, more than half of Cufflinks predictions reproduce AS events from the gene models. TAU uses known splice sites to predict all possible exons in a gene, generating vast numbers of novel exons and novel IR events.

### Comparison with TAU and Cufflinks

To see how SpliceGrapher's AS predictions compared with other tools, we ran the HashMatch/Supersplat/TAU and TopHat/Cufflinks pipelines, both with and without gene models, using the same RNA-Seq data as SpliceGrapher, and assembled splice graphs from their transcript predictions. We note that Cufflinks was designed for mammalian genomes: its assembly algorithm includes heuristics that are based on the characteristics of human and mouse transcripts [22]. TAU was designed to analyze the *A. thaliana *data we consider here [3].

Our first step in comparing SpliceGrapher to Cufflinks and TAU was to test the ability of each package to predict given and novel annotations. This was quantified by computing the fraction of exons and introns in the annotations that were predicted by each package (Equation 1 in the Materials and methods section); this is the 'recall' at the exon/intron level. These statistics are summarized in Table 2. First we discuss the rate at which each package is able to recall TAIR9 annotations. Without gene models, Cufflinks and TAU achieve similar levels of recall, which are below 0.29. This illustrates the difficulty of predicting transcriptional activity from NGS data alone. Recall improves dramatically when we provide Cufflinks and TAU with gene models: for Cufflinks it increases to 0.94 at the exon level, and to 0.89 at the intron level; for TAU it increases to 0.69 at the exon level, and 0.79 at the intron level. Since SpliceGrapher uses the input annotations as a baseline, it achieves perfect recall on TAIR9; Cufflinks and TAU sometimes ignore the annotations that are provided as input, which is a result of how they use annotations in their algorithms. Cufflinks, for example, breaks down annotated exons into pseudo-reads that are incorporated into its algorithm; and since it looks for a minimal set of transcripts that explain the observed read depth, the annotations can be ignored.

**Table 2:** Recall of TAIR9 and TAIR10 annotations

	TAIR9				TAIR10			
	
	Recall		Novel		Transcripts		Recall	
	
Method	Exons	Introns	Exons	Introns	Number	Percentage	Exons	Introns
**Splicegrapher**	1.00	1.00	1,428	1,282	28	1.4%	0.039	0.045
**Splicegrapher + EST**	1.00	1.00	11,299	3,557	38	1.9%	0.050	0.056
**Cufflinks**								
No gene models	0.25	0.19	33,252	3,425	0	0.0%	0.035	0.017
With gene models	0.94	0.89	12,690	5,222	4	0.2%	0.017	0.008
**TAU**								
No gene models	0.21	0.29	86,346	5,335	0	0.0%	0.043	0.029
With gene models	0.69	0.79	115,130	3,734	11	1.1%	0.079	0.074

Comparison of the ability of SpliceGrapher, Cufflinks, and TAU to predict TAIR9 and TAIR10 annotations in *A. thaliana*. The columns of the table provide recall levels of TAIR9 annotations, the number of novel exons and introns that are predicted, i.e., are not in the TAIR9 annotations, the number and percentage of TAIR10 transcripts that are predicted, and the recall level of TAIR10 annotations at the exon and intron level. When TAU and Cufflinks rely on RNA-Seq data alone they tend to produce graphs that are missing many of the features found in the gene models, as reflected in their recall scores between 0.19 and 0.29. When we provide them with TAIR9 annotations their recall scores improve, though TAU's improved statistics result from a vast number of novel exons, many of which may be spurious. When comparing these predictions with novel splice forms in the TAIR10 gene models, SpliceGrapher predicts more novel splice forms correctly than the other packages.

**Figure 4:**
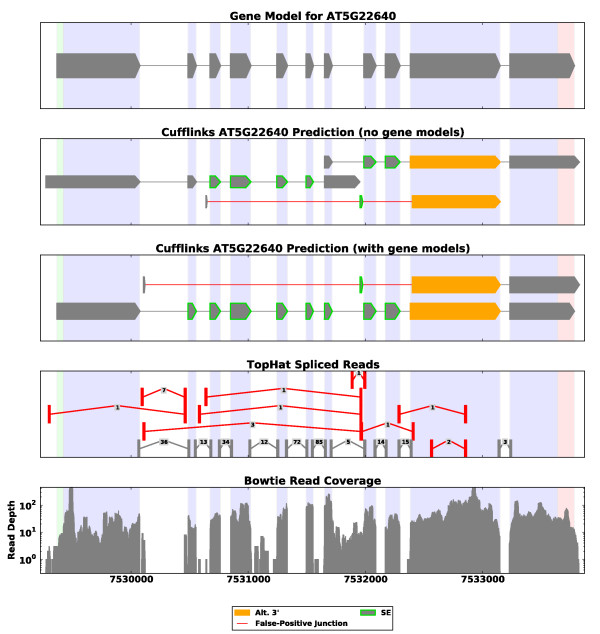
**Example of a Cufflinks prediction**. We provide the predictions made by Cufflinks for the same gene whose SpliceGrapher predictions are shown in Figure 1. Some of the splice junctions used by Cufflinks are predicted to be false positives by SpliceGrapher's accurate splice junction classifiers (red edges in the plot). These lead to detection of questionable AS events.

When TAU and Cufflinks use only RNA-Seq data, the transcripts that they predict are sometimes fragmented as a result of gaps in short-read coverage (Figure 4; Figures S1 to S3 in Additional file 1). For Cufflinks the number of genes for which it made predictions increased significantly when provided with gene models: from 10,277 genes to 28,277 in *A. thaliana *and from 10,022 to 23,634 in *V. vinifera*. For TAU the increase was not so dramatic, and when given gene models we have observed that it tends to predict what we believe are overly complex models. Figure S2 in Additional file 1 is a representative example.

None of the packages was able to predict more than 8% of the new annotations that were added in the TAIR10 version of the *Arabidopsis *genome annotations (see Table 2 for details). SpliceGrapher achieved better recall than Cufflinks-with or without annotations-despite making fewer overall predictions of new exons and introns; this is a strong indication that SpliceGrapher's predictions have fewer false positives. Surprisingly, the recall level for Cufflinks with gene models was lower than without them. SpliceGrapher also achieved better recall than TAU without annotations. With gene models, TAU achieved the highest recall at the exon and intron levels, but we believe it is a result of a severe over-annotation that was discussed earlier. At the transcript level SpliceGrapher performed best. Its splice graphs were consistent with 28 new splice forms, compared with 4 for Cufflinks and 11 for TAU when provided with TAIR9 annotations. Both packages were unable to correctly predict any transcript without using the gene models. In Additional file 1 we compare Cufflinks and SpliceGrapher predictions to results we have recently obtained using a curated set of full-length cDNAs and ESTs for SR genes in *Arabidopsis *[32].

#### Alternative splicing predictions

SpliceGrapher predicts AS events in proportions that more closely match the gene models than TAU and Cufflinks (Table 1). TAU uses known splice sites to predict all possible putative exons for a gene, resulting in a vast number of IR events that represent 85% of its AS predictions (Figure S2 in Additional file 1 demonstrates the issue). There is a noticeable discrepancy in the rate at which Cufflinks detects exon skipping events. Exon skipping accounts for 9% of the AS events in the gene models, whereas 43% of the novel AS events predicted by Cufflinks with gene models are ES. Detection of an exon skipping event requires a novel splice junction, and therefore depends on accurate splice junction detection. Both TAU and Cufflinks ignore the sequence characteristics of putative junctions, a practice that can easily lead to false positives. SpliceGrapher uses splice site classifiers to ensure the accuracy of reads that span splice junctions. We believe that the lower rate of exon skipping detected by SpliceGrapher is a result of better control of the quality of splice junction alignments. Below we discuss this issue in detail.

### Splice junction reads

We compared splice-junction read predictions made by the three packages. We restricted this comparison to junctions within known genes that were bounded by canonical GT-AG or GC-AG splice sites. Accurate splice junction alignments are crucial to the quality of AS predictions. This is illustrated in Figures S4 to S8 in Additional file 1. SpliceGrapher uses its splice-site classifiers to predict donor and acceptor sites that are then used to build a database of putative splice junction sequences. Short reads that align to these sequences provide evidence of splicing events recapitulated in the RNA-Seq data. Supersplat performs spliced alignment by attempting to map each end of a read to locations in the genome. It accepts alignments where a read's ends match genomic sequences with 100% identity and the inferred intron length is within specified limits. TopHat maps spliced reads by splitting the reads into segments, aligning the segments to genomic sequences, and accepting spliced alignments when they infer introns that are bounded by canonical GT-AG splice-site dimers and have lengths within specified limits.

Although the three packages find similar numbers of reads that span splice junctions, there is a sizable discrepancy in the number of novel splice junctions that were detected. For example, TopHat detected 14,572 novel splice junctions in *A. thaliana*. Only 1,982 of those were also detected by SpliceGrapher. Out of the 12,590 that were not detected by SpliceGrapher, 9,942 contained a putative splice site that was classified as a false-positive by our splice-site classifiers. Our classifiers achieve very high accuracy in *A. thaliana*, with an area under the receiver operating characteristic (ROC) curve between 0.95 and 0.97 for GT, GC, and AG sites (see Materials and methods section for details, and ROC curves in Figure S9 in Additional file 1). Therefore, the false positives identified in Table 3 for the alignments produced by TAU and Cufflinks are likely accurate.

**Table 3:** Comparison of splice junctions identified by each package

	SpliceGrapher	Supersplat	TopHat
*A. thaliana*			
Canonical junctions (GT-AG/GC-AG) within genes	80,421	84,744	83,367
Junctions in common	-	74,821	63,710
Novel junctions	4,969	7,255	14,572
Novel junctions with a false-positive site	-	3,077	9,942
Novel junctions in common	-	3,599	1,982
*V. vinifera*			
Canonical junctions (GT-AG/GC-AG) within genes	74,457	82,281	65,439
Junctions in common	-	70,554	59,154
Novel junctions	9,831	13,394	6,307
Novel junctions with a false-positive site	-	4,040	1,899
Novel junctions in common	-	8,020	3,662

Side-by-side comparison of canonical splice junctions identified by each package, reconciling differences between SpliceGrapher and the other two packages for *A. thaliana *(top half) and *V. vinifera *(bottom half). For each species we show the number of canonical splice junctions each package found recapitulated in the RNA-Seq reads. For Supersplat and TopHat we also show the number of junctions each shares with SpliceGrapher and the number of novel junctions for which SpliceGrapher's classifiers identified either the donor site or the acceptor site as a false positive.

Our use of a filtering step raises the concern that we are removing legitimate splice junctions, despite the high accuracy of the splice junction classifiers. As further validation, we compare the rate at which each package recapitulates splice junctions that are observed in a large collection of EST alignments. For *A. thaliana *the ESTs consisted of 1.5 million sequences from the National Center for Biotechnology Information (NCBI) dbEST database [37] and 71,806 sequences from [35], making a total of 1.6 million ESTs that aligned to 4,696 (16.5%) *A. thaliana *genes. For *V. vinifera *we downloaded 352,984 ESTs from the Plant Genome Database [38]. These ESTs aligned to 10,168 (43%) *V. vinifera *genes. The ESTs were aligned using a pipeline developed in [32], which uses GMAP [39] to align ESTs to a reference genome, and then assigns aligned ESTs to gene regions and corrects alignment artifacts. The results in Table 4 show that the three packages had a similar proportion of RNA-Seq junctions that matched junctions from the ESTs in both species. SpliceGrapher had the highest proportion of matches in *A. thaliana *and the second-highest in *V. vinifera*, indicating that its predictions were not adversely affected by the splice junction filtering step.

**Table 4:** Recall of splice junctions identified from EST data

Species	SpliceGrapher	Supersplat	TopHat
*A. thaliana*	24,757	25,590	22,934
*V. vinifera*	35,403	37,291	34,081

We compared the splice junctions that each package identified in RNA-Seq data to splice junctions inferred from ESTs aligned to *A. thaliana *and *V. vinifera*. Numbers represent the number of splice junctions from spliced alignments of RNA-Seq data that were also identified from alignments of ESTs (excluding junctions that are annotated in TAIR9). Despite performing a step of filtering of false positives, SpliceGrapher achieves a higher level of recall than TopHat, and only slightly lower than SuperSplat.

We next illustrate that spliced alignment filtering is important, even as read length increases. To do so, we generated 41 million 76-nucleotide reads for *A. thaliana *and created a set of shorter reads by truncating the 76-nucleotide reads to 32 nucleotides; we then ran TopHat on the two sets. We used the same TopHat parameters in both cases except for the segment length, which we set to 20 nucleotides for the 32-nucleotide reads and 26 nucleotides for the 76-nucleotide reads. The 76-nucleotide reads produced more than four times as many spliced alignments as their truncated counterparts; the shorter reads resulted in a slightly larger number of ungapped alignments since their full-length versions might span splice junctions (see Table S2 in Additional file 1 for details). The additional spliced alignments for 76-nucleotide reads increased the number of novel junctions more than six-fold, but the proportion of false-positive junctions also jumped from 24% to 39%. These results demonstrate the value of increased read length for sensitivity in splice junction detection, and yet longer read length alone does not guarantee accurate splice junction predictions.

Inaccurate spliced-alignment can have a strong impact on the accuracy of AS detection. To demonstrate this, we looked at the splice junctions predicted by TAU and Cufflinks and collected statistics for the AS events associated with those junctions that SpliceGrapher's classifiers identified as false positives (Table S3 in Additional file 1). We identified spurious splice junctions as those that contained at least one false-positive splice site. With gene models, 24% of the AS events predicted by Cufflinks could be attributed to spurious splice junctions, compared to 59% without gene models. Note that the percentages were computed with respect to AS events that are not in the TAIR9 annotations, so do not depend on the algorithm's access to the annotations. With TAU we also saw a decrease in the percentage of false positive events when provided with gene models.

Each of the three packages use somewhat different criteria for accepting splice-junction alignments. Both Supersplat and TopHat allow users to control maximum allowed intron length; SpliceGrapher on the other hand only accepts splice junctions that are within a single gene. We have found many examples where TAU and Cufflinks predicted splice junctions that span two genes; with TAU for example, we found 309 cases of splice junctions that span multiple genes in *A. thaliana *and 341 cases in *V. vinifera*.

### Incorporating ESTs

**Figure 5:**
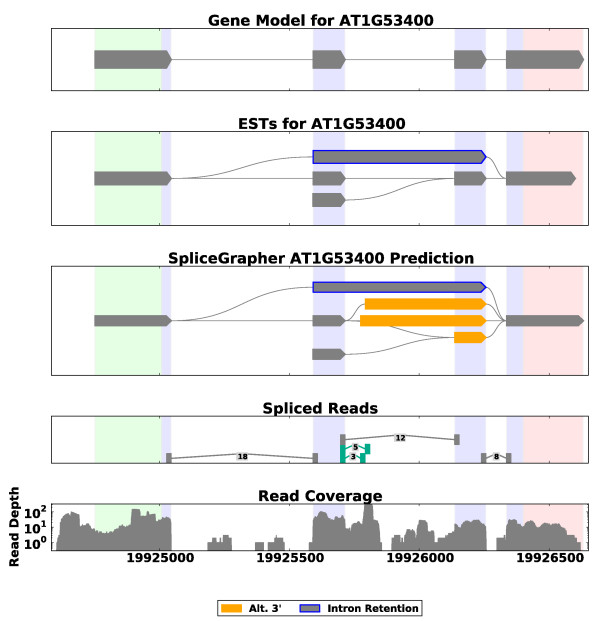
**SpliceGrapher prediction from RNA-Seq and EST data**. This example shows how SpliceGrapher can use both RNA-Seq data and EST data to produce predictions that incorporate the strengths of each data type. RNA-Seq data provide evidence for two novel splice junctions (fourth panel down, highlighted in green) that SpliceGrapher uses to infer an alternative 3' splicing event. EST alignments provide compelling evidence for an intron retention event. SpliceGrapher combines these predictions into the final predicted graph.

To demonstrate SpliceGrapher's ability to incorporate multiple forms of data into splice graph predictions, we ran it with the *A. thaliana *RNA-Seq data and provided the EST alignments described above. Figure 5 illustrates the benefit of combining RNA-Seq data and EST alignments for predicting splice graphs. In this example, ESTs provided evidence for an IR event that was not detected in the RNA-Seq data because read coverage was not consistent across the entire intron. RNA-Seq, on the other hand, provides evidence for alternative 3' splicing events. The predicted graph then incorporates the full range of evidence into a single coherent splice graph.

The addition of EST data increased the number of AS events from 7,388 to 9,916 and the number of genes where AS is observed from 4,901 to 6,162. The graphs augmented with EST alignment information contain novel AS events in proportions that were much closer to the proportions in the TAIR9 models than graphs predicted using RNA-Seq alone. Notably, the proportion of IR events is the same as in the gene models, demonstrating the utility of ESTs for detecting IR events. Finally, the level of recall of TAIR10 annotation has resulted in a large increase with the addition of the EST data (Table 2).

### Applying SpliceGrapher to mammalian genomes

To demonstrate SpliceGrapher's utility with mammalian genomes, we used a subset of the human RNA-Seq data used in [40] to compare AS in 60 individuals. We focused on the two individuals for whom a much larger set of reads was available. The data consisted of 74 million paired-end 35-nucleotide reads from two individuals: Caucasian (35.7 million reads) and Yoruban (38.3 million reads). ENSEMBL gene models were used as additional input to SpliceGrapher.

Based on *Homo sapiens *gene models, SpliceGrapher's classifiers achieved ROC scores of 0.97, 0.95 and 0.91 for GT, AG and GC splice sites, respectively. These classifiers predicted 25.3 million potential splice sites out of 105.2 million dimers that occurred within genes. Because of the longer gene length in human, the approach of creating a splice junction database was not feasible. Instead, we used TopHat to perform spliced alignments and filtered the results using our predicted splice site database.

We ran TopHat with default parameter settings to align both data sets. Our classifiers flagged close to 60% of the splice junctions in both datasets as false-positives (Table S4 in Additional file 1). This is in comparison to 14% in *Arabidopsis *and 58% in grape, which is further illustration of the potential pitfalls in splice junction alignment of short reads.

Using the filtered TopHat splice junction alignments, we found 1,099 novel AS events in the Caucasian sample and 1,154 novel AS events in the Yoruban sample. The original paper reported statistics on 110 AS events in genes where genetic variation impacted differential alternative expression, while our predictions span the whole genome. The various types of AS are predicted at rates similar to those in the gene models with the notable exception of IR, which was relatively hard to predict from plant RNA-Seq as well. The number of novel AS predictions in the two samples were similar for each AS type. Full details are provided in Table S5 in Additional file 1; Figure S10 in Additional file 1 shows an example where we identified a difference in the observed pattern of AS in the two samples.

## Conclusions

We have presented SpliceGrapher, a Python scripting package designed to enhance existing gene annotations by predicting splice graphs from RNA-Seq and EST data. In addition, SpliceGrapher includes modules for identifying AS events and for viewing predicted splice graphs along with the evidence used to generate them.

We compared SpliceGrapher to TAU and Cufflinks; unlike these packages, which make somewhat limited use of gene models, SpliceGrapher exploits gene annotations fully to construct its splice graphs. This allows SpliceGrapher to make meaningful predictions even for genes that have low read coverage, and helps us resolve AS events that are otherwise hard to detect from short-read data. Prediction of AS requires accurate alignment across splice junctions, which is a difficult task when read length is short. Our work demonstrates that using tools that do not use information about splice site characteristics can lead to a large number of possibly erroneous alignments. SpliceGrapher addresses this issue by using a machine learning approach to construct a database of potential splice sites that can be used to filter the output of a spliced alignment method such as TopHat, or to construct a database of potential splice junctions that allow the use of standard mapping tools that do not perform spliced alignment. The latter approach is only feasible for more compact genomes like the plant genomes we analyzed here.

We have demonstrated SpliceGrapher on RNA-Seq data from human and two plants, *A. thaliana *and *V. vinifera*. Plant genomes have different architectures from mammalian genomes and are therefore characterized by different types of AS events. In particular, IR is rare in mammalian genomes, but is the dominant form of AS in plants. Most existing work on predicting AS from short-read data has focused on exon skipping in mammals [1,18,19,41-44]. We have shown that SpliceGrapher is able to predict different forms of AS, although the rate at which it detects intron retention is lower than its observed frequency in existing annotations. This is due to the inherent difficulty in predicting intron retention from RNA-Seq data, which requires consistent read coverage across an entire intron.

## Materials and methods

### RNA-Seq data

In our experiments, we used publicly available short-read sequence data for the plants *A. thaliana *and *V. vinifera*. We downloaded FASTQ sequence files containing 284 million *A. thaliana *reads (accession number [SRA:009031]) and 59 million 36-nucleotide *V. vinifera *reads (accession number [SRX:012280]) from the NCBI Sequence Read Archive [45]. *A. thaliana *reads were trimmed to 32 nucleotides as recommended in [3]. As baseline annotation for *A. thaliana *we used the TAIR9 genome annotations [46]; the newer TAIR10 annotations were used for evaluation. For *V. vinifera *we used the Genoscope version 2 annotations and sequences, using only those sequences for the 19 best-characterized chromosomes. Additional RNA-Seq data were generated for *A. thaliana *as follows. Total RNA from 2-week-old *A. thaliana *(ecotype Columbia) seedlings grown on MS (Murashige and Skoog medium) plates was isolated using RNeasy Plant Mini Kit from Qiagen (Valencia, CA, USA). To remove any contaminating DNA, RNA was treated with DNAse [47]. Isolation of poly (A) mRNA and preparation of a cDNA library were carried out using the Illumina TrueSeq RNA kit. Sequencing (72 cycle) was done on an Illumina Genome Analyzer II. This dataset, which altogether has 41 million reads, has been deposited in NCBI's Gene Expression Omnibus [48], and is accessible through GEO series accession number [GSE:32318].

### The SpliceGrapher pipeline

SpliceGrapher's pipeline for predicting splice graphs uses several forms of data. Gene models are loaded directly into a splice graph that serves as a baseline for interpreting the RNA-Seq and EST data. Data from RNA-Seq experiments is aligned to the reference genome; ungapped alignment is carried out first, and all unaligned reads undergo spliced alignment, to infer reads that span splice junctions. SpliceGrapher can accept short-read alignments provided by a variety of tools [9-12,14-17]. EST and cDNA sequences are aligned using conventional sequence alignment tools [39,49] before SpliceGrapher includes the alignments in a splice graph. As SpliceGrapher loads each form of data, it integrates the new evidence with the gene models to construct a splice graph.

### Splice graph construction

Each of the data sources used by SpliceGrapher-genome annotations, short-read data, and EST alignments-requires a distinct interpretation for splice graph construction. For the following discussion of the procedures used to interpret each type of data we borrow terminology from [29] and refer to exons that have explicit acceptor and donor splice sites as 'bounded' and those with an undefined acceptor or donor splice site as 'unbounded'. When one graph element, such as an exon or an intron, falls completely within the genomic coordinates of another graph element, we say that it is 'contained' within the other element.

#### Gene models

SpliceGrapher accepts gene model annotations in GFF3 format [50] and constructs graphs using the sequence coordinates found in *UTR, CDS *and *exon *records. It interprets *exon *records as bounded exons and incorporates them directly into a splice graph. SpliceGrapher infers an intron whenever the corresponding exons are adjacent in a transcript. When an exon appears in multiple transcripts, a single exon node is created along with edges that link it to the exons that follow or precede it in the corresponding transcripts.

#### RNA-Seq data

To incorporate RNA-Seq data into a splice graph, SpliceGrapher loads read alignments in SAM format [51] for all reads that map within the boundaries of some annotated gene. It then constructs exons (hereafter referred to as 'short-read exons') from clusters of contiguous ungapped alignments where the read depth remains above a minimum threshold (the default is 0). A short-read exon is discarded if it is contained within an existing exon. If a short-read exon does not extend exactly to known or predicted acceptor or donor splice sites, SpliceGrapher will extend it to the nearest splice site that is known or has support from spliced reads.

#### EST alignments

Because they are longer than RNA-Seq reads, ESTs provide more reliable transcriptional evidence. In our framework we run EST alignments through a series of processing steps designed to remove alignment artifacts [32] and convert the alignments directly to splice-graphs using the same procedures we developed for gene models. Exons at the 3' or 5' end of an EST are considered unbounded. These are merged with other exons in the graph according to the following rules. An exon that is unbounded at its 3' end is merged with another exon if they share the same acceptor site and both exons are unbounded at the 3' end. If one of the exons is bounded, the exons will be merged only if the unbounded exon is contained within the bounded exon. Analogous rules apply to exons unbounded at the 5' end. SpliceGrapher infers an intron between exons whenever they are adjacent in an EST.

### Alternative splicing inference from RNA-Seq

Because of their short length, RNA-Seq data cannot be unambiguously interpreted as splice-graphs and AS events (Figure 3). SpliceGrapher's approach is to use as much data as possible to make confident predictions, and to annotate AS events as unresolved if the evidence does not clearly support a specific isoform. SpliceGrapher applies inference rules in the order presented in the following sections.

#### Intron retention

IR is arguably the most challenging form of AS to infer from RNA-Seq data. SpliceGrapher infers IR events from RNA-Seq evidence in two ways that exploit information from the gene models. When short-read coverage remains above a desired threshold across an intron's full length, it is evidence that the intron was retained in some transcripts (Figure 1). In this case the intron is excised in the constitutive form represented by the gene model. In an alternative scenario shown in Figure S11 in Additional file 1, the intron is retained in the known constitutive form. We detect this form of IR when a known exon has a novel splice junction within it.

When SpliceGrapher infers a novel IR event, it must identify unique exon boundaries for three exons: the longer exon in which the intron is retained, and the two shorter exons that flank the intron when it is excised. Usually the gene model provides good evidence for these boundaries, but in some cases it may not be possible to resolve them unambiguously.

When short-read coverage remains high across an intron's full length, SpliceGrapher will create a short-read exon that spans the intron. Its next task is to determine the exon's correct boundaries. SpliceGrapher first finds all exons in the graph that overlap the short-read exon. If one upstream exon overlaps its 5' end and one downstream exon overlaps its 3' end, SpliceGrapher creates a new exon whose boundaries are the acceptor site from the upstream exon and the donor site from the downstream exon. If more than one exon overlaps either end of the short-read exon, it is still possible to infer the new exon's boundaries provided all overlapping upstream exons share the same acceptor site and overlapping downstream exons share the same donor site (Figure S12 in Additional file 1). If the boundary at either end is ambiguous, SpliceGrapher creates an unresolved IR event. When a junction is used to infer an IR event through the scenario of a splice junction within an exon, SpliceGrapher must identify the boundaries for two new exons, which is performed in a manner analogous to the single exon case.

In some cases read coverage may remain high across two or more introns in succession, making it impossible to determine which of several possible splice forms is correct (Figure 3). In these cases, SpliceGrapher annotates the corresponding short-read exon as unresolved.

#### Alternative 3' and 5' events

When an intron is excised at more than one splice site, changing the boundaries of one of its flanking exons, we have evidence of an alternative 3' or 5' event. SpliceGrapher uses two forms of evidence to infer alternative 3'/5' events. When a short-read exon overlaps an existing exon but extends beyond its 3' or 5' end, it provides evidence for an alternative donor or acceptor site. In addition, when a novel splice junction appears between two exons it provides evidence for a novel intron (Figure S12 in Additional file 1). SpliceGrapher requires both forms of evidence to infer a novel alternative splice site. Below we describe the procedure for inferring an alternative acceptor (3' site). The procedure for an alternative donor (5' site) is analogous.

When a short-read exon overlaps an existing exon and extends into its upstream intron, it is evidence that the exon boundaries changed in some transcripts. To identify an acceptor site for the new exon, SpliceGrapher looks for junctions that have acceptor sites within the same intron, upstream of the short-read exon (Figure S12 in Additional file 1). SpliceGrapher then uses the acceptor site nearest the short-read exon as its acceptor site. If it finds no acceptor sites within the intron, SpliceGrapher annotates the short-read exon as unresolved.

If SpliceGrapher can resolve a new exon's acceptor site, it must resolve its donor site as well. The procedure is the same as that for identifying retained intron boundaries in the previous section. If one downstream exon overlaps the new exon's 3' end, SpliceGrapher uses the downstream exon's donor site as the new exon's donor site. If more than one downstream exon overlaps the new exon's 3' end, SpliceGrapher can still resolve its donor site provided all overlapping exons share the same donor site (Figure S12 in Additional file 1). If the overlapping exons have different donor sites, SpliceGrapher cannot resolve the new exon's donor, and it annotates the exon as unresolved.

#### Exon skipping

An exon skipping event occurs when an exon is excised from some transcripts but included in others. SpliceGrapher infers skipped exons in two different ways. In the first scenario the exon is included in the known constitutive form represented in the gene model. If a novel splice junction spans the existing exon, it is evidence that the exon was skipped in some transcripts (see top panel of Figure S13 in Additional file 1). If the novel junction's acceptor and donor sites match those of established exons, SpliceGrapher adds the new intron to the graph and annotates the skipped exon.

An alternative scenario is when the exon is skipped in the constitutive form (bottom panel of Figure S13 in Additional file 1). In this case, if a short-read exon falls within an intron and is flanked by two novel junctions, it is evidence for a novel exon that is skipped in some transcripts. These clues may not provide enough evidence to resolve the event, so SpliceGrapher tries to associate the upstream junction's donor site and the downstream junction's acceptor site with exons in the graph. If this first step is successful, SpliceGrapher uses the upstream junction's acceptor site as the new exon's acceptor site and the downstream junction's donor site as the new exon's donor site. If it is unable to resolve the junctions, SpliceGrapher annotates the short-read exon as unresolved.

### Splice graph assembly

The above rules are used to infer exons from gene models, ESTs and short-read data. The final step consists of adding edges (introns) that connect consecutive exons. For each newly predicted exon, SpliceGrapher looks for other exons in the graph that use the same acceptor site. If any of these exons has an intron from its acceptor site to a neighboring exon, SpliceGrapher adds an edge from the neighboring exon to the new exon. It repeats the process for all exons that have the same donor site. SpliceGrapher uses an analogous procedure to infer introns for the donor site of a new exon. Graphs are stored using a GFF file format.

### Splice graph comparisons

TAU and Cufflinks predict transcripts that we convert into splice graphs associated with annotated genes. As *de novo *prediction tools, both TAU and Cufflinks make predictions without regard to known gene boundaries. Thus, to perform a meaningful comparison we associate each transcript with one or more genes that it overlaps. Once transcripts are associated with annotated genes, they are converted to splice graphs using the same procedure SpliceGrapher uses to assemble splice graphs from ESTs.

In order to compare a predicted splice graph with the splice graph generated from genome annotations, we compare the number of exons and introns from the gene model that are recapitulated. This is quantified using the 'recall' statistic. The recall of a set *A *with respect to a set *B *is defined as:

$$R\;\left(A|B\right)=\frac{A\cap B}{B}$$

We define *R*(*A*|*B*) = 1 when *B *is the empty set. To compare two splice graphs we compute recall for exons and introns separately.

### Short read alignment

SpliceGrapher includes modules that allow it to incorporate RNA-Seq data into splice graph predictions. It accepts short read alignments in SAM format [51]. For this project we used PASS [10] to perform short-read alignments as we found it to be fast and accurate in preliminary tests on synthetic data.

To provide high-confidence splice graph predictions, we enforced strict criteria for accepting alignments. We accepted only reads that aligned to the genomic reference with 100% identity at a unique location. To increase confidence in these alignments, we also required that a read align nowhere else in the genome at 90% identity or above (a procedure adapted from [13]). We used uniquely aligned reads to identify exonic regions, and unaligned reads for splice-junction alignments.

### Splice junction reads

SpliceGrapher performs spliced alignment by first constructing a database of splice junction sequences that are formed by concatenating the sequence directly upstream of a donor site with the sequence directly downstream of an acceptor site. We distinguish three kinds of splice junctions: 'known' junctions that are derived from gene model annotations; 'recombined' junctions constructed from novel combinations of known splice sites; and 'predicted' junctions in which one or both splice sites are novel. SpliceGrapher constructs junction sequences by pairing every known and predicted donor site in a gene with all known and predicted acceptor sites downstream of it in the same gene. This procedure has been used in other studies (see, for example, [1,18,19]) to construct a database of known and recombined splice junctions from known splice sites. SpliceGrapher extends this procedure to include predicted sites as well.

To avoid spurious alignments, we require that junction-crossing reads align with a minimum 10-nucleotide overlap on either side of a junction. Adopting nomenclature from [14], we refer to the 10-nucleotide region on either side of a junction as the 'anchor' region. For reads of length *n *and a required overlap size *p*, we can enforce this constraint by generating splice junction sequences of length 2(*n-p*). For example, for 32-nucleotide reads and a required 10-nucleotide minimum overlap, we generated 44-nucleotide sequences (22 nucleotides on either side of a junction). To improve sensitivity, we accepted alignments with 90% identity overall, provided they aligned with 100% identity within the anchor region.

The three pipelines each used slightly different alignment criteria. Our SpliceGrapher pipeline allowed mismatches in exonic alignments so that we could identify and eliminate reads that aligned well to multiple locations. We also accepted mismatches in splice-junction alignments to improve sensitivity, provided there were no mismatches within anchor regions. We used TopHat parameters that allowed us to duplicate our own alignment criteria, though TopHat accepts mismatches within splice-junction anchor regions. Supersplat accepts only reads that align with 100% identity within anchor regions.

### Splice site prediction

We classify splice sites using support vector machines, using an approach that has been shown to be highly accurate in splice site prediction [12,15,52,53]. To create splice site classifiers, SpliceGrapher extracts positive and negative example sequences for splice site donor and acceptor dimmers such as GT, GC and AG following the procedure described in [52]. Briefly, we use known splice sites as positive examples for a given dimer and for negative examples we use all other occurrences of the dimer found within genes. Training examples then consist of intronic and exonic sequences taken from either side of a site.

SpliceGrapher's classifiers discriminate sequences using an implementation of the weighted-degree kernel [52]. This kernel represents a sequence in a feature space of k-mers associated with positions in the sequence. Kernel parameters include exon and intron sequence lengths on either side of a splice site, k-mer length, number of mismatches to allow within a k-mer, and whether to allow shifts in k-mer position (for a detailed overview, see [54]). SpliceGrapher iterates over combinations of these parameters to identify the best-performing combination. It uses the PyML package [55] to train and test these support vector machines using the training examples described above. SpliceGrapher executes this procedure for all given acceptor and donor dimers to yield a classifier for each one. It then applies each classifier to corresponding dimers in the genomic reference sequences. Locations classified as splice sites contribute a pool of predicted sites that SpliceGrapher combines with known splice sites when it generates splice junction sequences. The Palmapper package [15] uses a similar set of classifiers, but evaluates acceptor and donor sites during spliced alignment instead of creating a splice junction database.

In predicting splice sites we focused on canonical GT and GC donor sites and AG acceptor sites. For *A. thaliana*, we used SpliceGrapher with TAIR9 annotations to extract 122,534 annotated GT, GC and AG splice sites. SpliceGrapher created splice site classifiers for GT and GC donor sites and AG acceptor sites. These classifiers achieved ROC scores of 0.97, 0.97 and 0.95 for GT, GC and AG sites, respectively, in five-fold cross-validation. ROC curves are provided in Figure S9 in Additional file 1. These classifiers predicted novel splice sites for 878,994 out of 9,753,440 dimers (9%) found within genes in the TAIR9 reference sequences. Combining these predicted sites with known splice sites, we generated a splice junction database with 8.2 million junctions, out of which 122,534 are known and 586,704 are recombined.

For *V. vinifera*, SpliceGrapher extracted 125,080 annotated GT, GC and AG splice sites from the gene models. The splice-site classifiers achieved ROC scores of 0.91, 0.81 and 0.88 for GT, GC and AG sites, respectively, in five-fold cross-validation. As *V. vinifera *is not as well annotated as *A. thaliana*, we decided to use the EST alignments described earlier to obtain better splice site models. We used SpliceGrapher to extract 84,811 splice sites from these alignments, and used them to improve our splice site models. The resulting classifiers achieved ROC scores of 0.98, 0.92 and 0.96 for GT, GC and AG sites, respectively, considerably higher than those based on the gene models. The ROC curves are shown in Figure S9 in Additional file 1. These classifiers predicted 2.2 million putative splice sites out of 11.2 million dimers (20%) within genes in the reference sequences. The resulting splice junction database contains 91 million splice junction sequences, out of which 125,080 are known, and 616,314 are recombined.

### HashMatch/Supersplat/TAU

We ran HashMatch and Supersplat [17] on the two data sets, following the procedures outlined in [3]. We first used HashMatch to perform ungapped read alignment. We then perform spliced alignment with Supersplat using those reads that did not result in a match in ungapped alignment. Supersplat performs alignment without regard for splice site dimers, so for a more realistic comparison we accepted only alignments that spanned canonical dimers. Supersplat accepts minimum and maximum intron lengths and anchor region size. It is prudent to select conservative intron sizes to prevent TAU from generating an inordinate number of transcripts. For *A. thaliana *we established an intron size range of 40 to 5,000, as 99.9% of known introns fell within this range. For *V. vinifera *the same criterion yielded an intron size range of 55 to 15,000. To fix a lower bound for overlaps on either side of a junction, we set the minimum anchor size to 10, and accepted only reads that aligned to a unique splice junction. All experiments used Supersplat version 1.0 and TAU version 1.4 (no version information was available for HashMatch).

### BowTie/TopHat/Cufflinks

TopHat and Cufflinks were designed for mammalian genomes and thus rely on some heuristics that are based on mammalian gene expression statistics and gene architecture. For example, TopHat's heuristic filter for spliced alignments is based on the observation that, in humans, minor splice forms usually have expression levels that are at least 15% as high as those of their corresponding major splice forms [14]. Another heuristic embedded into TopHat is to report only alignments across GT-AG introns for reads shorter than 75 nucleotides.

We ran TopHat on the *A. thaliana *data using parameters that reflected the requirements we set for our own alignments: no multi-hits (-g 1), minimum anchor length 10 (-a 10), and minimum and maximum intron length 40 and 5,000, respectively (55 and 15,000 for *V. vinifera*). TopHat splits reads into segments for part of its search. To permit reads to align at 90% identity, we set the segment length to 20 (--segment-length = 20) and allowed up to 2 mismatches per segment (--segment-mismatches = 2). To eliminate the heuristic filter associated with mammalian genomes, we set the minimum normalized depth to 0 (-F 0). To make our comparison as fair as possible, we also ran TopHat with --segment-mismatches = 0 to force it to accept only alignments with 100% identity. We then approximated our own alignment criteria by using TopHat's ungapped alignments at 100% identity and its spliced alignments at 90% identity. The remaining difference between TopHat's splice-junction alignments and ours was a subset of TopHat read alignments that contained mismatches in the anchor region. All experiments used Bowtie version 4.1.2, TopHat version 1.3.3 and Cufflinks version 1.1.0.

## Abbreviations

AS: alternative splicing; CDS: coding sequence; ES: exon skipping; EST: expressed sequence tag; GFF: general feature format; IR: intron retention; NCBI: National Center for Biotechnology Information; NGS: next-generation sequencing; ROC: receiver operating characteristic; SAM: sequence alignment/map; TAIR: The *Arabidopsis *Information Resource; UTR: untranslated region.

## Authors' contributions

This project was conceived by AB and ASNR, and carried out by MFR under the supervision of AB. JT carried out the experiments that provided additional RNA-Seq data, supervised by ASNR. All authors read and approved the final manuscript for publication.

## Supplementary Material

Additional file 1 **Supplementary information**. Includes a section on validating SpliceGrapher predictions with evidence from other experiments, plus supplementary tables and figures. Click here for file

## References

[B1] Mortazavi A Williams B McCue K Schaeffer L Wold B Mapping and quantifying mammalian transcriptomes by RNA-Seq. Nat Methods 2008 5 621 628 10.1038/nmeth.1226 18516045 PMC13303166

[B2] Wang Z Gerstein M Snyder M RNA-Seq: a revolutionary tool for transcriptomics. Nat Rev Genet 2009 10 57 63 10.1038/nrg2484 19015660 PMC2949280

[B3] Filichkin S Priest H Givan S Shen R Bryant D Fox S Wong W Mockler T Genome-wide mapping of alternative splicing in Arabidopsis thaliana. Genome Res 2010 20 45 10.1101/gr.093302.109 19858364 PMC2798830

[B4] Harr B Turner L Genome-wide analysis of alternative splicing evolution among Mus subspecies. Mol Ecol 2010 19 228 239 20331782 10.1111/j.1365-294X.2009.04490.x

[B5] Ramani A Calarco J Pan Q Mavandadi S Wang Y Nelson A Lee L Morris Q Blencowe B Zhen M Fraser A Genome-wide analysis of alternative splicing in Caenorhabditis elegans. Genome Res 2011 21 342 10.1101/gr.114645.110 21177968 PMC3032936

[B6] Stamm S Ben-Ari S Rafalska I Tang Y Zhang Z Toiber D Thanaraj T Soreq H Function of alternative splicing. Gene 2005 344 1 20 15656968 10.1016/j.gene.2004.10.022

[B7] Hallegger M Llorian M Smith CWJ Alternative splicing: global insights. FEBS J 2010 277 856 866 10.1111/j.1742-4658.2009.07521.x 20082635

[B8] Shendure J Ji H Next-generation DNA sequencing. Nat Biotechnol 2008 26 1135 1145 10.1038/nbt1486 18846087

[B9] Li H Ruan J Durbin R Mapping short DNA sequencing reads and calling variants using mapping quality scores. Genome Res 2008 18 1851 10.1101/gr.078212.108 18714091 PMC2577856

[B10] Campagna D Albiero A Bilardi A Caniato E Forcato C Manavski S Vitulo N Valle G PASS: a program to align short sequences. Bioinformatics 2009 25 967 10.1093/bioinformatics/btp087 19218350

[B11] Langmead B Trapnell C Pop M Salzberg S Ultrafast and memory-efficient alignment of short DNA sequences to the human genome. Genome Biol 2009 10 R25 10.1186/gb-2009-10-3-r25 19261174 PMC2690996

[B12] De Bona F Ossowski S Schneeberger K Rätsch G Optimal spliced alignments of short sequence reads. BMC Bioinformatics 2008 9 O7 10.1186/1471-2105-9-S10-O7 18689821

[B13] Yassour M Kaplan T Fraser H Levin J Pfiffner J Adiconis X Schroth G Luo S Khrebtukova I Gnirke A Nusbaum C Thompson D Friedman N Regev A Ab initio construction of a eukaryotic transcriptome by massively parallel mRNA sequencing. Proc Natl Acad Sci USA 2009 106 3264 10.1073/pnas.0812841106 19208812 PMC2638735

[B14] Trapnell C Pachter L Salzberg S TopHat: discovering splice junctions with RNA-Seq. Bioinformatics 2009 25 1105 1111 10.1093/bioinformatics/btp120 19289445 PMC2672628

[B15] Jean G Kahles A Sreedharan V Bona F Rätsch G RNA-Seq Read Alignments with PALMapper. Curr Protocols Bioinformatics 2010 32 11.6.1 11.6.37 10.1002/0471250953.bi1106s32 21154708

[B16] Wang K Singh D Zeng Z Coleman S Huang Y Savich G He X Mieczkowski P Grimm S Perou C MacLeod J Chiang D Prins J Liu J MapSplice: Accurate mapping of RNA-seq reads for splice junction discovery. Nucleic Acids Res 2010 38 e178 10.1093/nar/gkq622 20802226 PMC2952873

[B17] Bryant D Shen R Priest H Wong W Mockler T Supersplat-spliced RNA-seq alignment. Bioinformatics 2010 26 1500 10.1093/bioinformatics/btq206 20410051 PMC2881391

[B18] Pan Q Shai O Lee L Frey B Blencowe B Deep surveying of alternative splicing complexity in the human transcriptome by high-throughput sequencing. Nat Genet 2008 40 1413 1415 10.1038/ng.259 18978789

[B19] Sultan M Schulz M Richard H Magen A Klingenhoff A Scherf M Seifert M Borodina T Soldatov A Parkhomchuk D Schmidt D O'Keeffe S Haas S Vingron M Lehrach H Yaspo M A global view of gene activity and alternative splicing by deep sequencing of the human transcriptome. Science 2008 321 956 959 10.1126/science.1160342 18599741

[B20] Wang E Sandberg R Luo S Khrebtukova I Zhang L Mayr C Kingsmore S Schroth G Burge C Alternative isoform regulation in human tissue transcriptomes. Nature 2008 456 470 476 10.1038/nature07509 18978772 PMC2593745

[B21] Tang F Barbacioru C Wang Y Nordman E Lee C Xu N Wang X Bodeau J Tuch B Siddiqui A Lao K Surani M mRNA-Seq whole-transcriptome analysis of a single cell. Nat Methods 2009 6 377 382 10.1038/nmeth.1315 19349980

[B22] Trapnell C Williams B Pertea G Mortazavi A Kwan G van Baren M Salzberg S Wold B Pachter L Transcript assembly and quantification by RNA-Seq reveals unannotated transcripts and isoform switching during cell differentiation. Nat Biotechnol 2010 28 511 515 10.1038/nbt.1621 20436464 PMC3146043

[B23] Guttman M Garber M Levin J Donaghey J Robinson J Adiconis X Fan L Koziol M Gnirke A Nusbaum C Rinn J Lander E Regev A Ab initio reconstruction of cell type-specific transcriptomes in mouse reveals the conserved multi-exonic structure of lincRNAs. Nat Biotechnol 2010 28 503 510 10.1038/nbt.1633 20436462 PMC2868100

[B24] Grabherr M Haas B Yassour M Levin J Thompson D Amit I Adiconis X Fan L Raychowdhury R Zeng Q Chen Z Mauceli E Hacohen N Gnirke A Rhind N di Palma F Birren B Nusbaum C Lindblad-Toh K Friedman N Regev A Full-length transcriptome assembly from RNA-Seq data without a reference genome. Nat Biotechnol 2011 29 644 652 10.1038/nbt.1883 21572440 PMC3571712

[B25] Simpson J Wong K Jackman S Schein J Jones S Birol I ABySS: a parallel assembler for short read sequence data. Genome Res 2009 19 1117 10.1101/gr.089532.108 19251739 PMC2694472

[B26] Heber S Alekseyev M Sze S Tang H Pevzner P Splicing graphs and EST assembly problem. Bioinformatics 2002 18 181 188 10.1093/bioinformatics/18.suppl_1.s181 12169546

[B27] Xing Y Resch A Lee C The multiassembly problem: reconstructing multiple transcript isoforms from EST fragment mixtures. Genome Res 2004 14 426 10.1101/gr.1304504 14962984 PMC353230

[B28] Sammeth M Valiente G Guigo R Bubbles: alternative splicing events of arbitrary dimension in splicing graphs. Lecture Notes Comput Sci 2008 4955 372 10.1007/978-3-540-78839-3_32

[B29] Harrington E Bork P Sircah: a tool for the detection and visualization of alternative transcripts. Bioinformatics 2008 24 1959 10.1093/bioinformatics/btn361 18635569

[B30] Bonizzoni P Mauri G Pesole G Picardi E Pirola Y Rizzi R Detecting alternative gene structures from spliced ESTs: a computational approach. J Comput Biol 2009 16 43 66 10.1089/cmb.2008.0028 19119993

[B31] Labadorf A Link A Rogers M Thomas J Reddy A Ben-Hur A Genome-wide analysis of alternative splicing in Chlamydomonas reinhardtii. BMC Genomics 2010 11 114 10.1186/1471-2164-11-114 20163725 PMC2830987

[B32] Richardson D Rogers M Labadorf A Ben-Hur A Guo H Paterson A Reddy A Comparative analysis of serine/arginine-rich proteins across 27 eukaryotes: insights into subfamily classification and extent of alternative splicing. PLoS ONE 2011 6 e24542 10.1371/journal.pone.0024542 21935421 PMC3173450

[B33] Zenoni S Ferrarini A Giacomelli E Xumerle L Fasoli M Malerba G Bellin D Pezzotti M Delledonne M Characterization of transcriptional complexity during berry development in Vitis vinifera using RNA-Seq. Plant Physiol 2010 152 1787 10.1104/pp.109.149716 20118272 PMC2850006

[B34] Reddy A Alternative splicing of pre-messenger RNAs in plants in the genomic era. Annu Rev Plant Biol 2007 58 267 294 10.1146/annurev.arplant.58.032806.103754 17222076

[B35] Wang B Brendel V Genomewide comparative analysis of alternative splicing in plants. Proc Natl Acad Sci USA 2006 103 7175 10.1073/pnas.0602039103 16632598 PMC1459036

[B36] Kim E Magen A Ast G Different levels of alternative splicing among eukaryotes. Nucleic Acids Res 2007 35 125 10.1093/nar/gkm529 17158149 PMC1802581

[B37] Boguski M Lowe T Tolstoshev C dbEST-database for "expressed sequence tags". Nat Genet 1993 4 332 333 10.1038/ng0893-332 8401577

[B38] PlantGDB http://plantgdb.org/

[B39] Wu T Watanabe C GMAP: a genomic mapping and alignment program for mRNA and EST sequences. Bioinformatics 2005 21 1859 10.1093/bioinformatics/bti310 15728110

[B40] Montgomery S Sammeth M Gutierrez-Arcelus M Lach R Ingle C Nisbett J Guigo R Dermitzakis E Transcriptome genetics using second generation sequencing in a Caucasian population. Nature 2010 464 773 777 10.1038/nature08903 20220756 PMC3836232

[B41] Blencowe B Ahmad S Lee L Current-generation high-throughput sequencing: deepening insights into mammalian transcriptomes. Genes Dev 2009 23 1379 10.1101/gad.1788009 19528315

[B42] Huang W Khatib H Comparison of transcriptomic landscapes of bovine embryos using RNA-Seq. BMC Genomics 2010 11 711 10.1186/1471-2164-11-711 21167046 PMC3019235

[B43] Wang L Xi Y Yu J Dong L Yen L Li W A statistical method for the detection of alternative splicing using RNA-Seq. PLoS ONE 2010 5 e8529 10.1371/journal.pone.0008529 20072613 PMC2798953

[B44] Richard H Schulz M Sultan M Nürnberger A Schrinner S Balzereit D Dagand E Rasche A Lehrach H Vingron M Haas S Yaspo M Prediction of alternative isoforms from exon expression levels in RNA-Seq experiments. Nucleic Acids Res 2010 38 e112 10.1093/nar/gkq041 20150413 PMC2879520

[B45] NCBI Sequence Read Archive. http://www.ncbi.nlm.nih.gov/sra

[B46] Swarbreck D Wilks C Lamesch P Berardini T Garcia-Hernandez M Foerster H Li D Meyer T Muller R Ploetz L Radenbaugh A Singh S Swing V Tissier C Zhang P Huala E The Arabidopsis Information Resource (TAIR): gene structure and function annotation. Nucleic Acids Res 2008 36 D1009 17986450 10.1093/nar/gkm965 PMC2238962

[B47] Palusa S Ali G Reddy A Alternative splicing of pre-mRNAs of Arabidopsis serine/arginine-rich proteins: regulation by hormones and stresses. Plant J 2007 49 1091 10.1111/j.1365-313X.2006.03020.x 17319848

[B48] Barrett T Troup DB Wilhite SE Ledoux P Evangelista C Kim IF Tomashevsky M Marshall KA Phillippy KH Sherman PM Muertter RN Holko M Ayanbule O Yefanov A Andrey Soboleva NCBI GEO: archive for functional genomics data sets-10 years on. Nucleic Acids Res 2011 39 D1005 D1010 10.1093/nar/gkq1184 21097893 PMC3013736

[B49] Kent W BLAT-the BLAST-like alignment tool. Genome Res 2002 12 656 11932250 10.1101/gr.229202 PMC187518

[B50] Eilbeck K Mungall C Lewis S Ashburner M The Sequence Ontology Project 2009. http://www.sequenceontology.org/gff3.shtml

[B51] Li H Handsaker B Wysoker A Fennell T Ruan J Homer N Marth G Abecasis G Durbin R The sequence alignment/map format and SAMtools. Bioinformatics 2009 25 2078 10.1093/bioinformatics/btp352 19505943 PMC2723002

[B52] Rätsch G Sonnenburg S Schölkopf B, Tsuda K, Vert JP Accurate splice site detection for Caenorhabditis elegans. Kernel Methods in Computational Biology 2004 MIT Press 277

[B53] Rätsch G Sonnenburg S SchÄolkopf B RASE: recognition of alternatively spliced exons in C. elegans. Bioinformatics 2005 21 i369 i377 10.1093/bioinformatics/bti1053 15961480

[B54] Ben-Hur A Ong C Sonnenburg S Schölkopf B Rätsch G Support vector machines and kernels for computational biology. PLoS Comput Biol 2008 4 e1000173 10.1371/journal.pcbi.1000173 18974822 PMC2547983

[B55] PyML-machine learning in Python. http://pyml.sourceforge.net/

