# Supplementary material for: SpliceGrapher: detecting patterns of alternative splicing from RNA-Seq data in the context of gene models and EST data
Source: Genome Biol. 2012 Jan 31;13(1):R4. doi: 10.1186/gb-2012-13-1-r4 (PMC3334585; doi:10.1186/gb-2012-13-1-r4)
Supplement: Additional file 1 — Supplementary information. Includes a section on validating SpliceGrapher predictions with evidence from other experiments, plus supplementary tables and figures. [file gb-2012-13-1-r4-S1.PDF]

Supplementary information for:

SpliceGrapher: detecting patterns of alternative splicing from RNA-seq data in the context of gene models and EST data

Mark F. Rogers[1], Julie Thomas[3] , Anireddy S.N. Reddy[3] , Asa Ben-Hur*[1,2]

[1]Department of Computer Science, Colorado State University, Fort Collins, Colorado, USA
[2]Department of Statistics, Colorado State University, Fort Collins, Colorado, USA
[3]Department of Biology and Program in Cell and Molecular Plant Biology, Colorado State University, Fort Collins, CO, USA

Email: Mark F. Rogers - rogersma@cs.colostate.edu; Julie Thomas - thomasju@lamar.colostate.edu; Anireddy S.N. Reddy - reddy@colostate.edu; Asa Ben-Hur*- asa@cs.colostate.edu;

*Corresponding author

- **SR gene case-study**
- **Supplementary Tables 1-5**
- **Supplementary Figures 1-17**

1 Validation with SR Genes

To further validate our SpliceGrapher method, we compared its predictions on 15 *A. thaliana* serine-arginine (SR) genes with results from a separate study [32]. Briefly, the authors obtained full-length genomic sequences for these genes from TAIR, and used MEGABLAST to find matching ESTs in NCBI's dbEST database and full-length cDNAs (fl-cDNA) in the nr nucleotide database. These sequences were fed along with genomic sequences into an in-house alignment pipeline to assess the extent of AS among the SR genes. We then used SpliceGrapher to produce predictions for these genes, using the two Arabidopsis short-read datasets described in the Methods section of the main text. We compared SpliceGrapher's predictions with the graphs produced from the EST and fl-cDNA alignments. Supplementary Figures 14-17 show the predictions made by SpliceGrapher and Cufflinks for SR33 (AT1G55310) using the 32nt and 76nt reads. The full set of output graphs are available at http://combi.cs.colostate.edu/SpliceGrapher/results/at_sr_genes.php. Despite having less reads, SpliceGrapher is able to make more predictions using the 76nt reads, and those predictions are in agreement with the predictions made from ESTs and fl-cDNAs. Although it is clear from the read coverage of the 76nt reads that there is an IR event, SpliceGrapher was not able to unambiguously determine which intron was retained, so no prediction was made (Figure 15).

We compare SpliceGrapher's predictions with those of Cufflinks on these same genes using the same data and supplying Cufflinks with the gene models in each case. Using the 32nt data with strong coverage across the gene (Supplementary Figure 16), Cufflinks predicts some of the same novel AS events as in the ESTs, but it also predicts an exon-skipping event that is not evident in the gene models, the RNA-Seq data or in the ESTs. With the 76nt data (Supplementary Figure 17), Cufflinks predicts only the gene models despite having the same evidence for a novel exon and IR events as SpliceGrapher.

We note that there is a splice junction in the EST/fl-cDNA graph that is marked as a false-positive site. The acceptor dimer for this exon is a non-canonical AT site and thus could not be predicted with our AG splice site classifier. Neither SpliceGrapher nor Cufflinks found evidence for this splice site. Cufflinks uses a heuristic that looks for GT-AG and GC-AG sites, while SpliceGrapher uses dimer-specific classifiers to predict splice sites. At this time the gene models and EST data we have for *A. thaliana* provide only 17 examples of AT sites, too few to train an accurate AT classifier.

2 Tables

Source	AS Genes	Intron Retention		Exon Skipping		Alt. 5'		Alt. 3'		Total
TAIR9	4,029	1,987	(33%)	550	(9%)	1,256	(21%)	2,145	(36%)	5,938
SpliceGrapher	5,173	2,503	(30%)	701	(8%)	1,769	(21%)	3,312	(40%)	8,285
novel	1,154	560	(23%)	151	(6%)	513	(21%)	1,167	(49%)	2,391

Supplementary Table 1: SpliceGrapher predictions from the 76nt RNA-Seq data generated in house. Despite generating fewer reads, improvements in NGS sequencing techniques yield longer, high-quality reads that make it easier to predict novel AS. As a result, SpliceGrapher was able to make confident predictions for more novel AS events of all types than with the older, 32nt data. The fraction of each type of event is similar in both cases. (Alt. 3'=alternative 3' site; Alt. 5'=alternative 5' site)

Relationship Between Read Length and False-Positive Rate						
Read Length	Total Alignments	Ungapped	Spliced	Total Junctions	Novel Junctions	FP (%)
32 nt	31,474,618	30,718,012	756,606	46,485	1,803	428 (23.7%)
76 nt	30,828,836	27,271,200	3,557,636	87,891	11,544	4,490 (38.9%)

Supplementary Table 2: The effect of read length on false positive splice junctions. We ran TopHat on 41,428,058 RNA-Seq reads in *A. thaliana*. Two TopHat runs were performed: on the original 76nt long reads, and on reads generated by truncating the reads to 32nt long. For the 76nt reads, the number of spliced alignments increased dramatically. Many of these were associated with novel splice junctions; however the false-positive rate was substantially higher than that of the 32nt reads. (RNA-Seq=RNA sequences produced by next-generation sequencing)

AS Associated with False-Positive Sites				
	Exon Skipping	Alt. 5'	Alt. 3'	Total
Cufflinks (no gene models)				
A. thaliana	267 (71.4%)	233 (62.8%)	215 (46.7%)	715 (59.3%)
V. vinifera	105 (20.2%)	66 (23.8%)	146 (21.1%)	317 (21.3%)
Cufflinks (with gene models)				
A. thaliana	327 (12.8%)	291 (50.5%)	278 (48.7%)	896 (24.2%)
V. vinifera	1,445 (43.3%)	2,036 (52.3%)	2,447 (48.1%)	5,928 (48.1%)
TAU (no gene models)				
A. thaliana	486 (77.0%)	978 (52.1%)	1,080 (34.3%)	2,544 (44.9%)
V. vinifera	169 (22.4%)	604 (24.7%)	1,400 (27.3%)	2,173 (26.1%)
TAU (with gene models)				
A. thaliana	915 (50.0%)	1,551 (26.2%)	1,543 (16.4%)	4,009 (23.4%)
V. vinifera	5,709 (67.1%)	7,060 (23.5%)	8,188 (25.9%)	20,828 (29.9%)

Supplementary Table 3: SpliceGrapher's classifiers identified many Cufflinks and TAU splice sites as false-positives. Above is shown the number and proportion of each novel AS event type for which predictions were influenced by false-positive sites. IR events do not contribute to these statistics because the packages do not depend on splice sites to resolve them.
(Alt. 3'=alternative 3' site; Alt. 5'=alternative 5' site)

TopHat Alignments for *H. sapiens* Data						
	Total Reads	Aligned Reads	Ungapped Alignments	Spliced Alignments	Novel Junctions	False-Positive Junctions
Caucasian	35,687,594	23,283,623	21,085,632	2,197,991	7,705	4,549
Yoruban	38,302,542	25,707,194	23,424,639	2,357,294	8,576	4,960

Supplementary Table 4: Summary of TopHat alignments for the paired-end *H. sapiens* data. SpliceGrapher's classifiers were used to construct a database of predicted splice sites that were then used to filter out false-positive junctions from TopHat alignments. Above is shown the total number of reads used in the alignments, the number of those that aligned, how they broke down into ungapped and spliced alignments, the number of novel splice junctions inferred by the alignments, and the number of false-positive junctions.

Alternative Splicing Statistics for 20,640 *H. sapiens* Graphs								
Source	Intron Retention		Exon Skipping		Alt. 5'		Alt. 3'		Total
Gene Models	11,853	(14%)	42,124	(50%)	15,342 (18%)	15,401 (18%)	84,720
Caucasian	11,944	(14%)	42,699	(50%)	15,515 (18%)	15,624 (18%)	85,782
Novel	124	(11%)	575	(52%)	174 (16%)	226 (21%)	1,099
Yoruban	11,960	(14%)	42,653	(50%)	15,561 (18%)	15,657 (18%)	85,831
Novel	142	(12%)	529	(46%)	221 (19%)	262 (23%)	1,154

Supplementary Table 5: Summary of AS events detected in the two *H. sapiens* data sets. Novel events were predicted at nearly the same rate as they appear in the gene models, with the exception of intron retention, which is difficult to predict from RNA-Seq data.
(Alt. 3'=alternative 3' site; Alt. 5'=alternative 5' site)

3 Figures

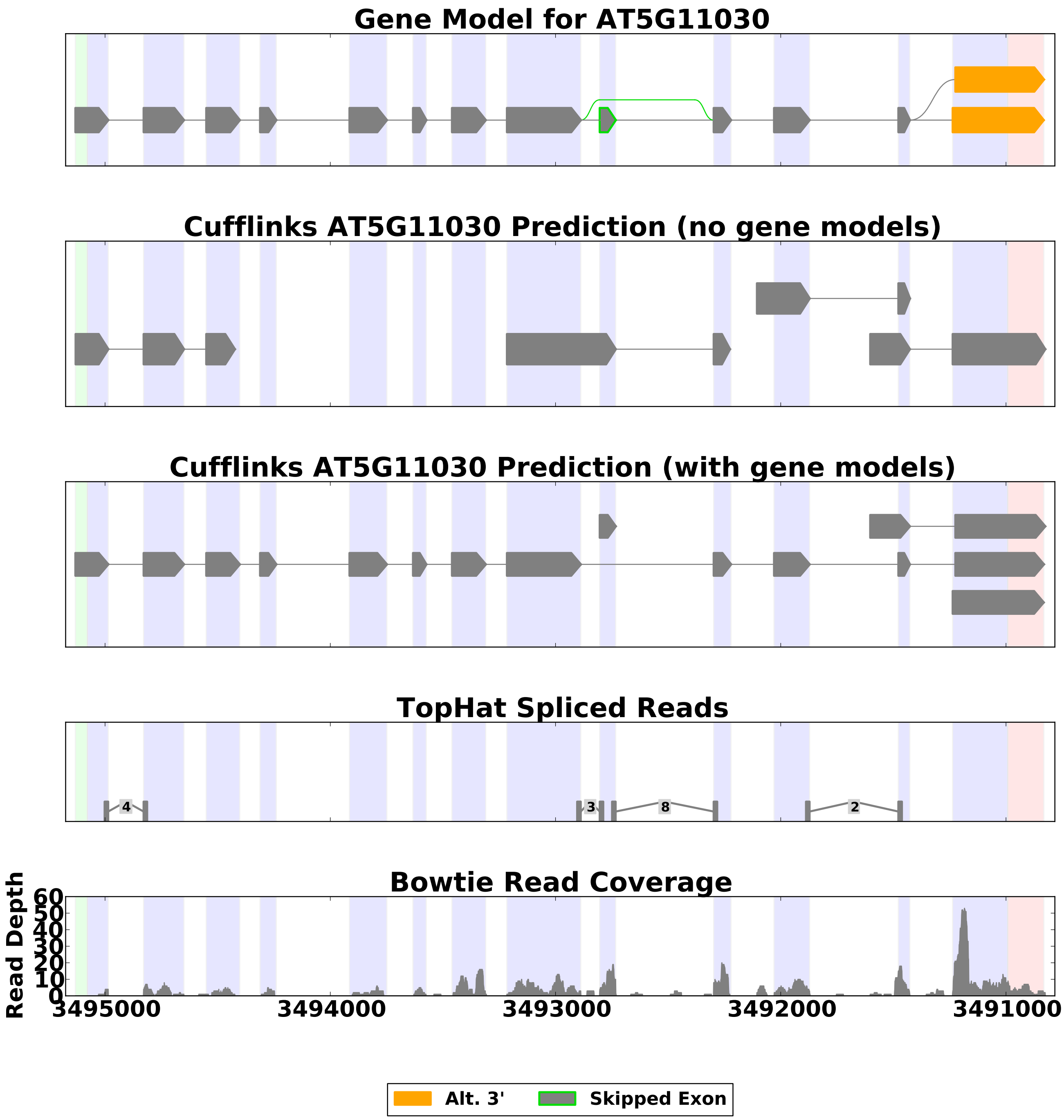

Supplementary Figure 1: Cufflinks predictions for the AT5G11030 gene from *A. thaliana* are both fragmented due to the low read coverage. (Alt. 3'=alternative 3' site)

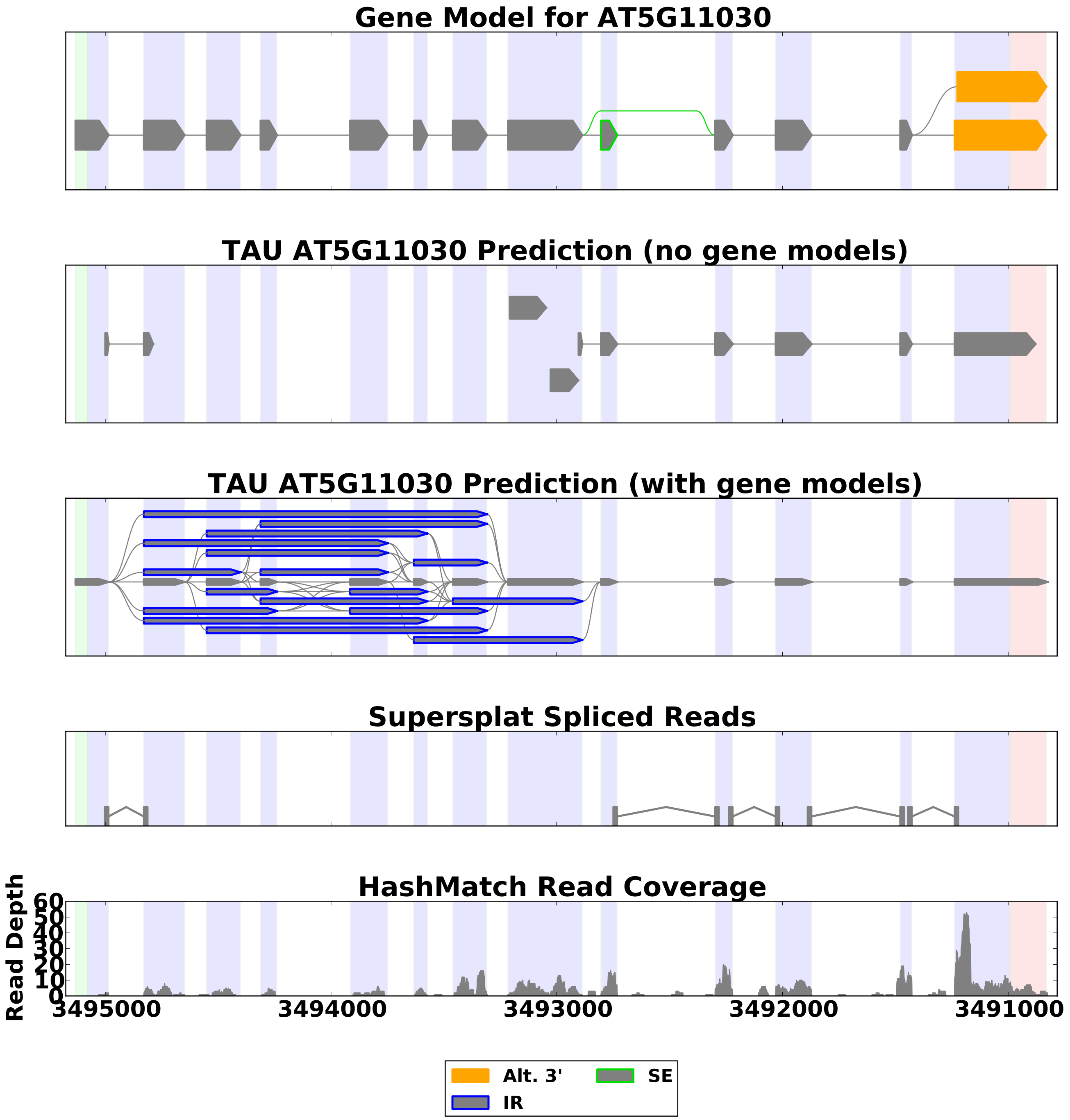

Supplementary Figure 2: TAU predicts several fragments for the AT5G11030 gene from *A. thaliana* when it has no gene models to work with, but predicts a large number of possibly spurious intron retention events when gene models are provided. (Alt. 3'=alternative 3' site; IR=intron retention, SE=skipped exon)

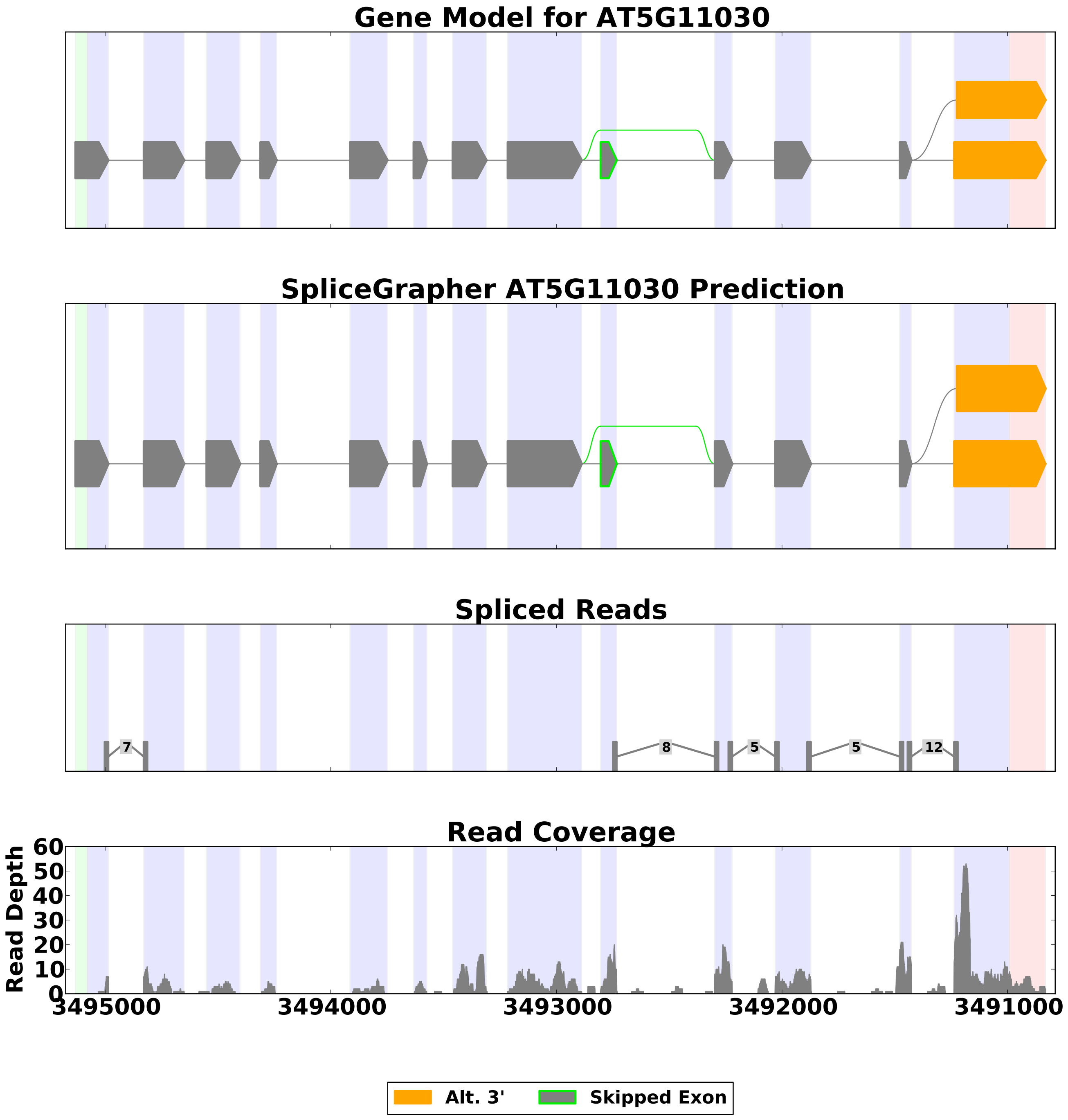

Supplementary Figure 3: SpliceGrapher prediction for the AT5G11030 gene, where low RNA-Seq coverage makes it challenging to predict AS. By using the gene model to establish a context for interpreting these data, SpliceGrapher is able to report the original gene model while still avoiding spurious predictions. (Alt. 3'=alternative 3' site)

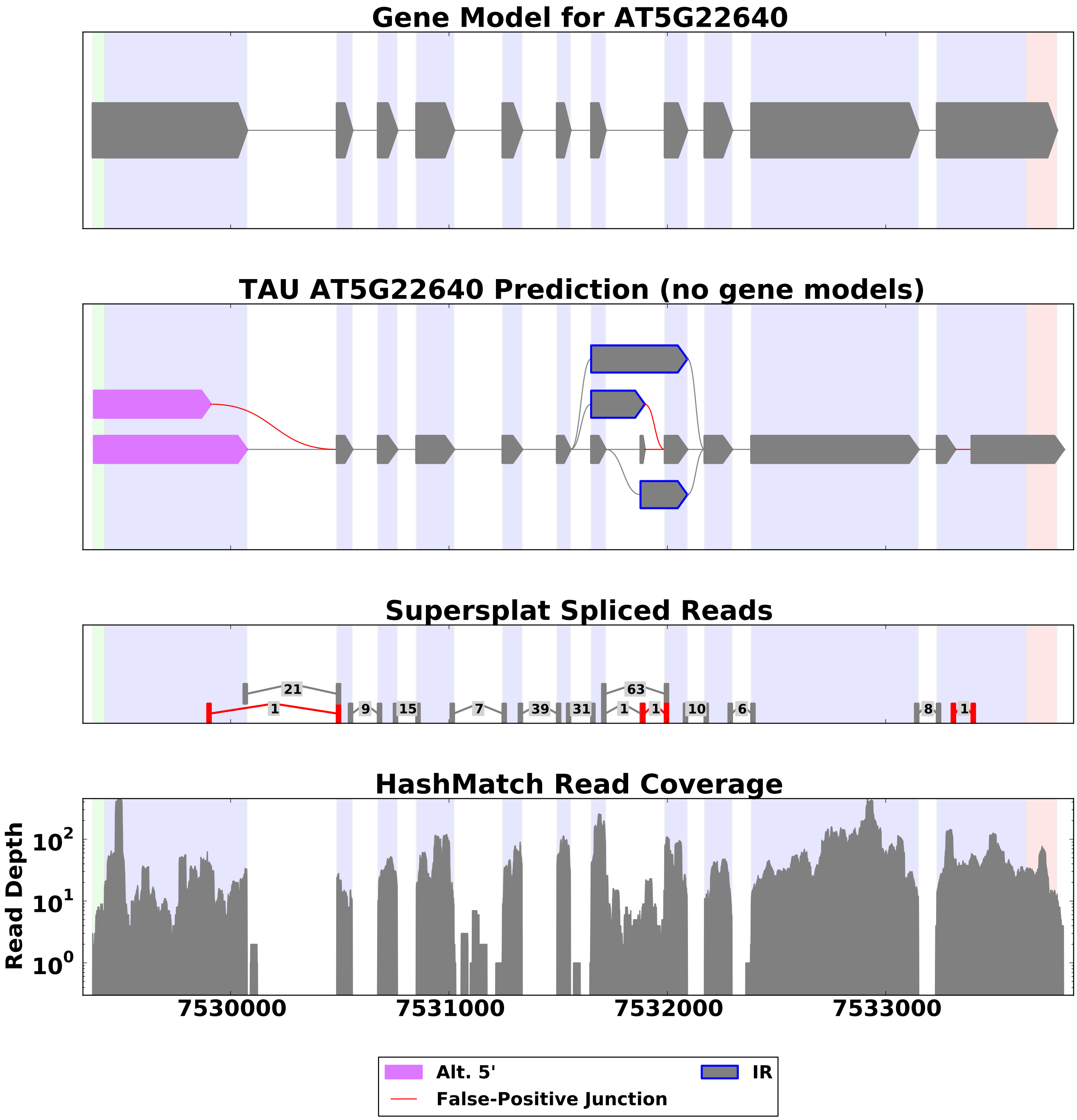

Supplementary Figure 4: TAU prediction along with the corresponding alignments for the *A. thaliana* gene AT5G22640. In this gene TAU did not produce any transcript predictions when we provided it with gene models. (Alt. 5'=alternative 5' site, IR=intron retention)

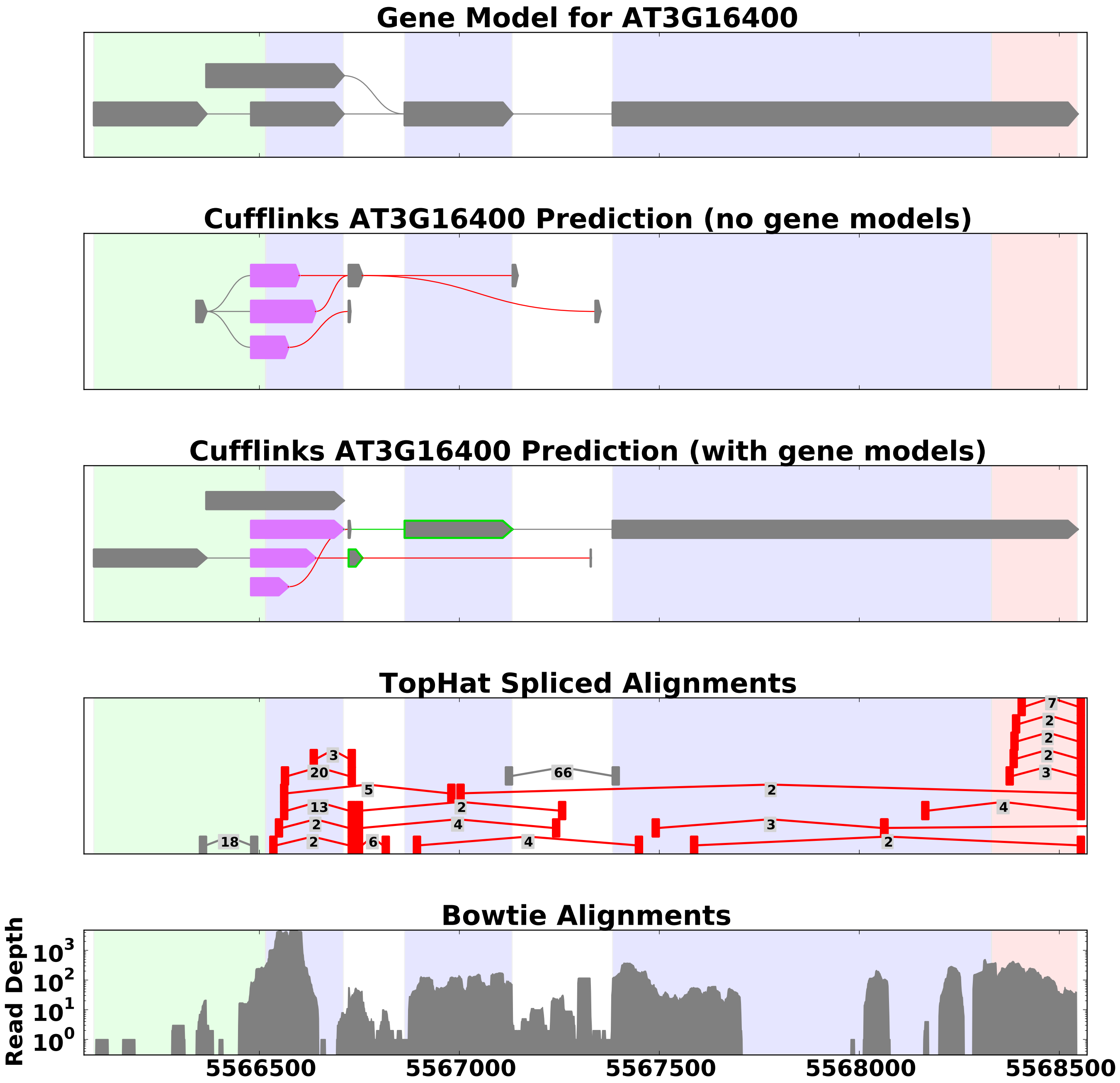

Supplementary Figure 5: Cufflinks prediction for the gene AT3G16400 in *A. thaliana*. Cufflinks attempts to find a set of transcripts that are consistent with the preponderance of splice-junction evidence. The resulting graph likely contains spurious splice forms.

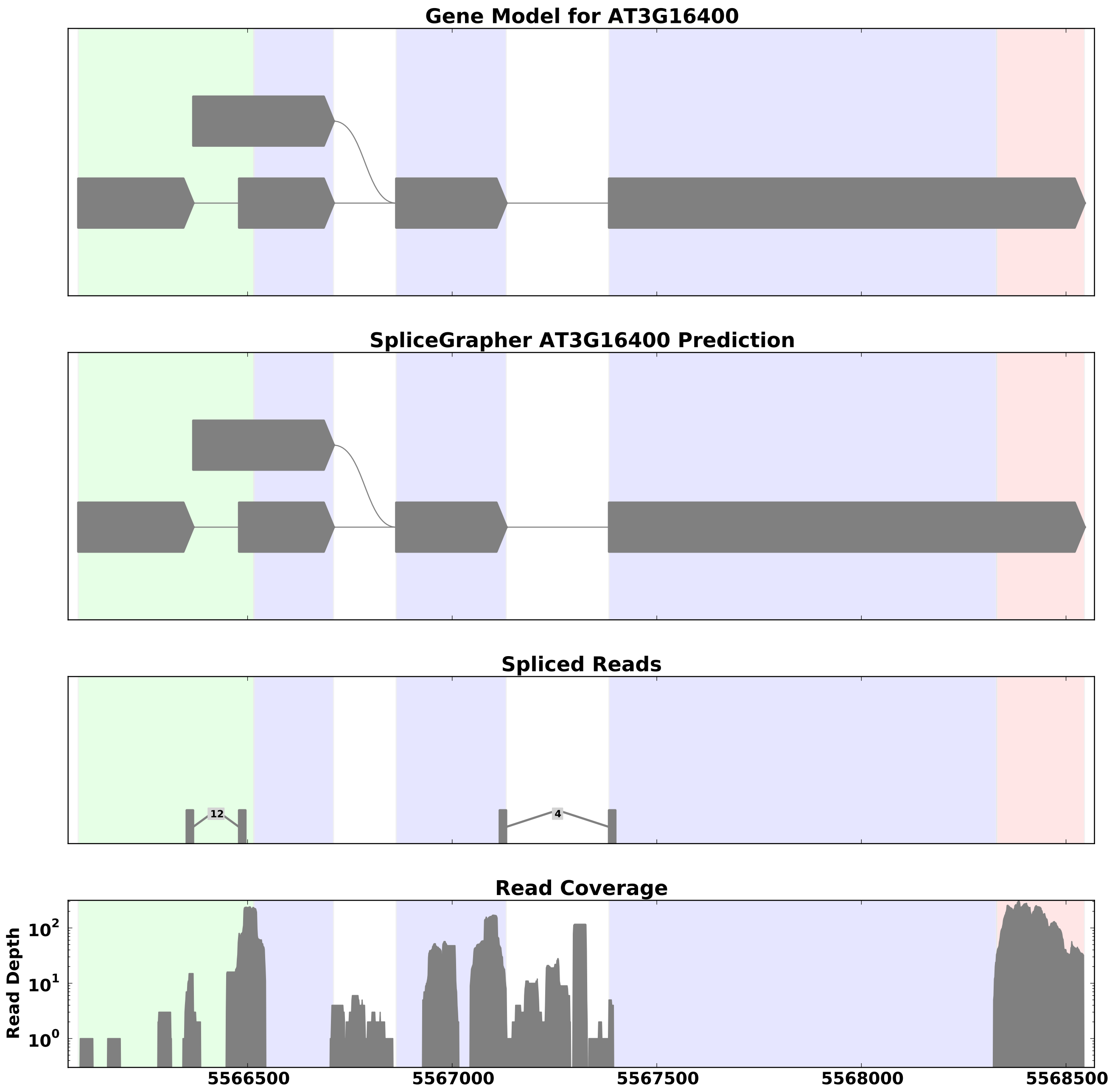

Supplementary Figure 6: SpliceGrapher predictions for the same gene where Cufflinks predicted many false positive splice-junctions. By first filtering out false-positive splice junction alignments, SpliceGrapher is able to avoid making spurious predictions.

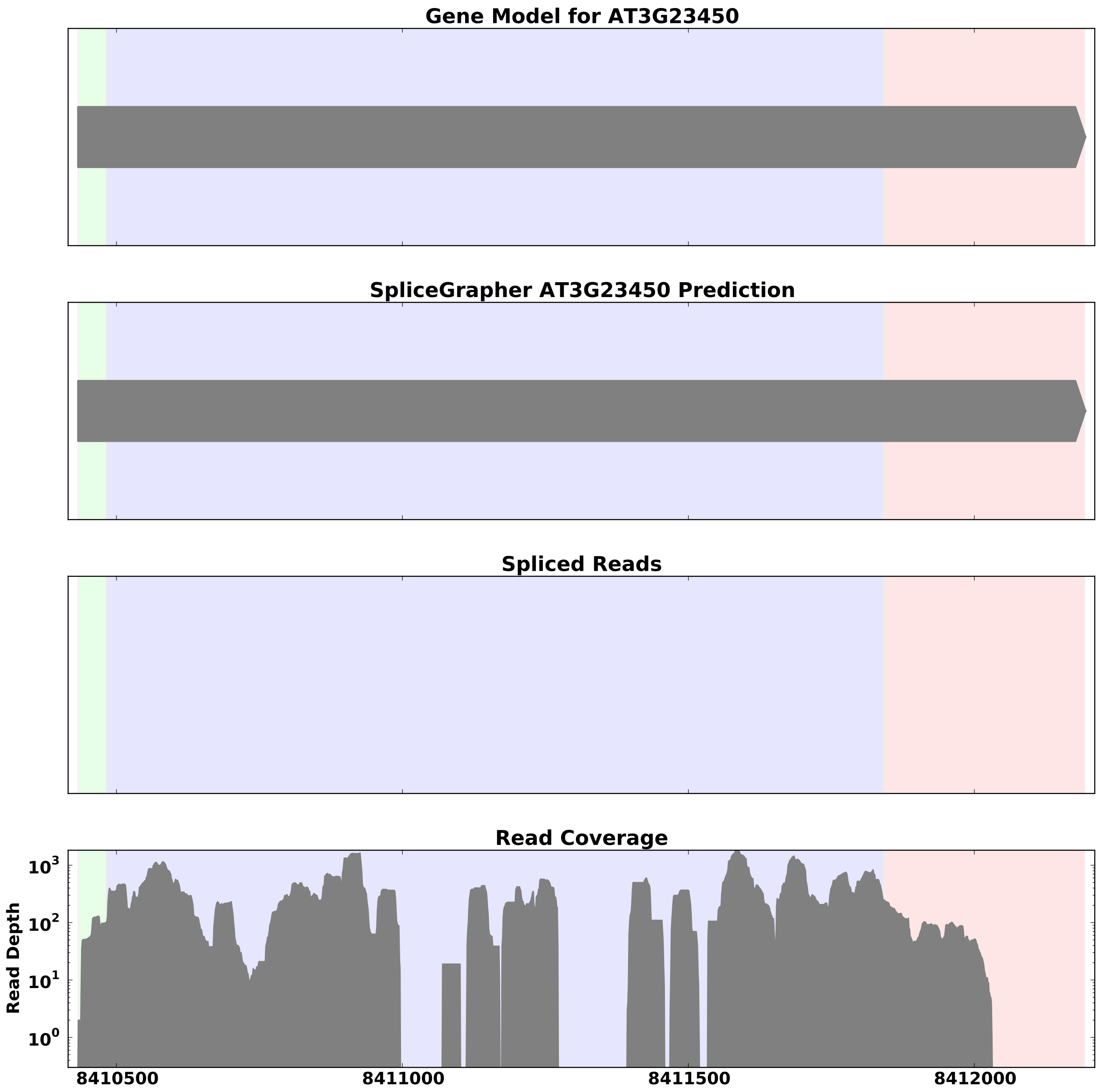

Supplementary Figure 7: SpliceGrapher prediction for a single-exon gene in *A. thaliana* where the data suggest novel alternative splicing but lack the splice junction evidence necessary for making confident predictions.

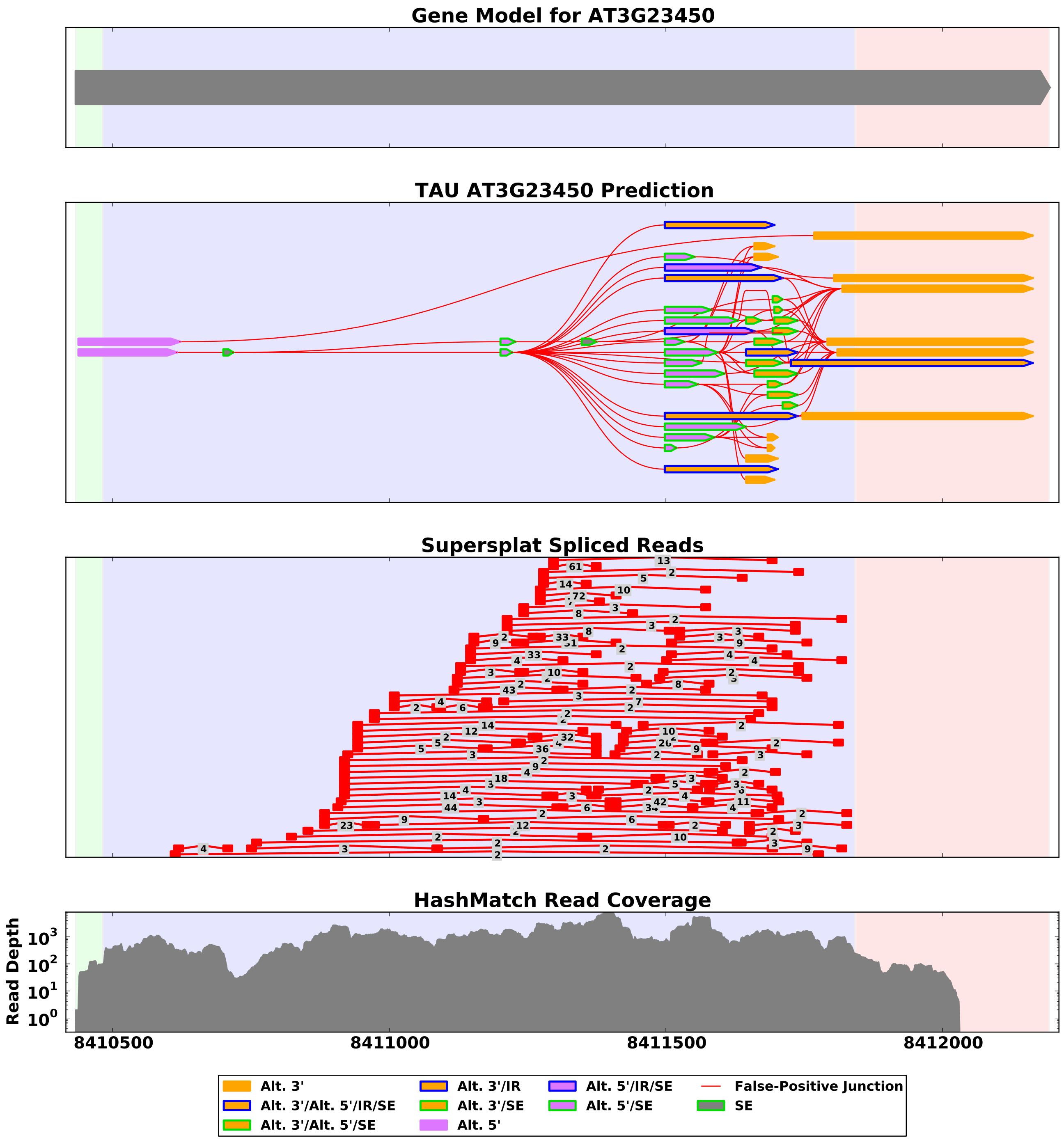

Supplementary Figure 8: TAU prediction for the AT3G23450 gene in *A. thaliana*. TAU attempts to find all transcripts that are consistent with the preponderance of splice-junction evidence. The resulting graph is likely to contain a substantial number of spurious splice forms. (Alt. 3'=alternative 3' site; Alt. 5'=alternative 5' site; IR=intron retention, SE=skipped exon)

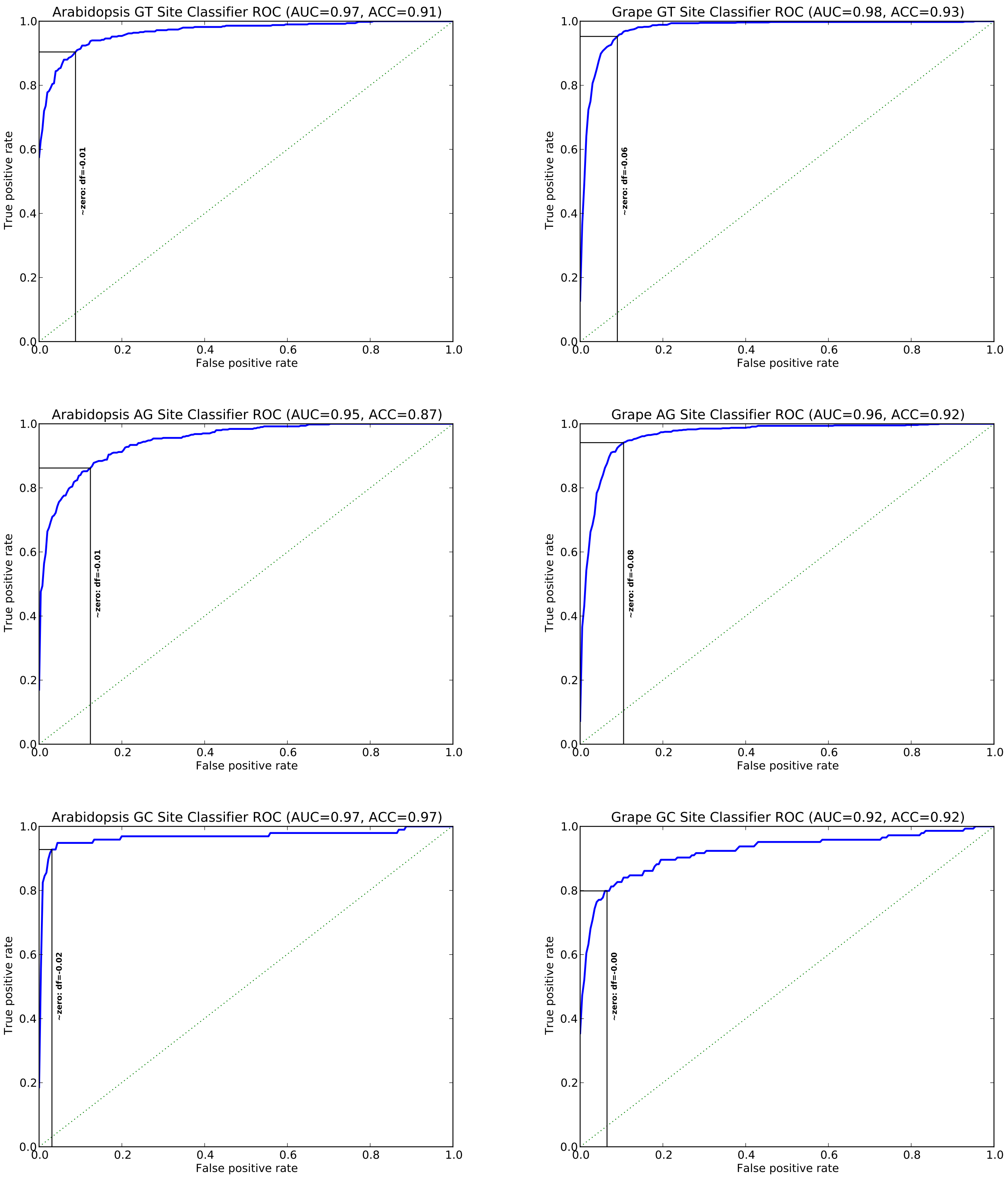

Supplementary Figure 9: ROC curves that demonstrate the accuracy of the SVM classifiers SpliceGrapher generated for GT and GC donor sites and AG acceptor sites in *A. thaliana* and *V. vinifera*. (ACC=accuracy; AUC=area under the curve; ROC=receiver operating characteristic, SVM=support vector machine)

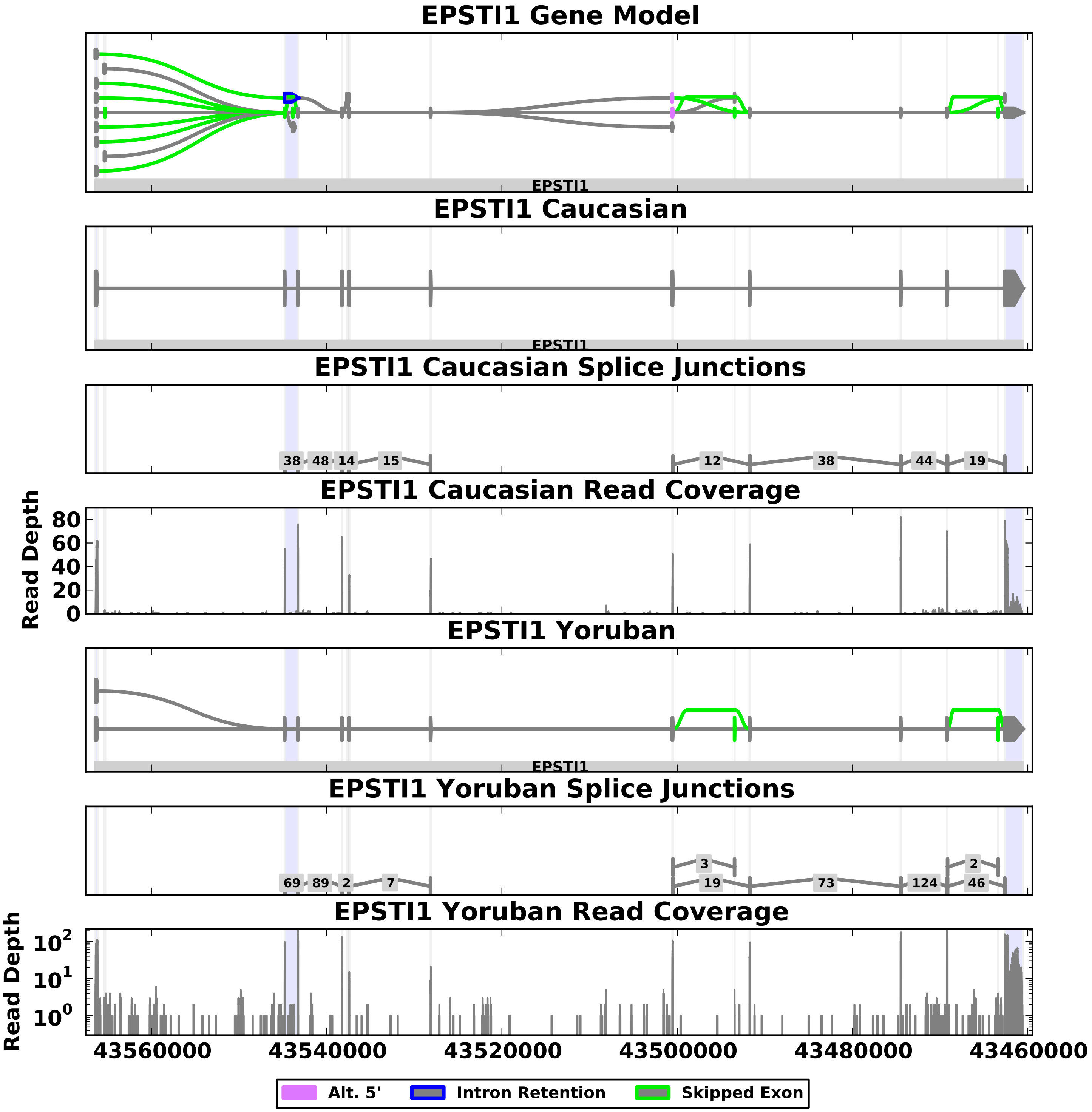

Supplementary Figure 10: Splice graphs for the human gene *EPSTI1* using short read data for Caucasian and Yoruban samples. Short-read coverage for exons and splice junctions identified a splice form in the Yoruban data that did not appear in the Caucasian data for this gene. (Alt. 5'=alternative 5' site)

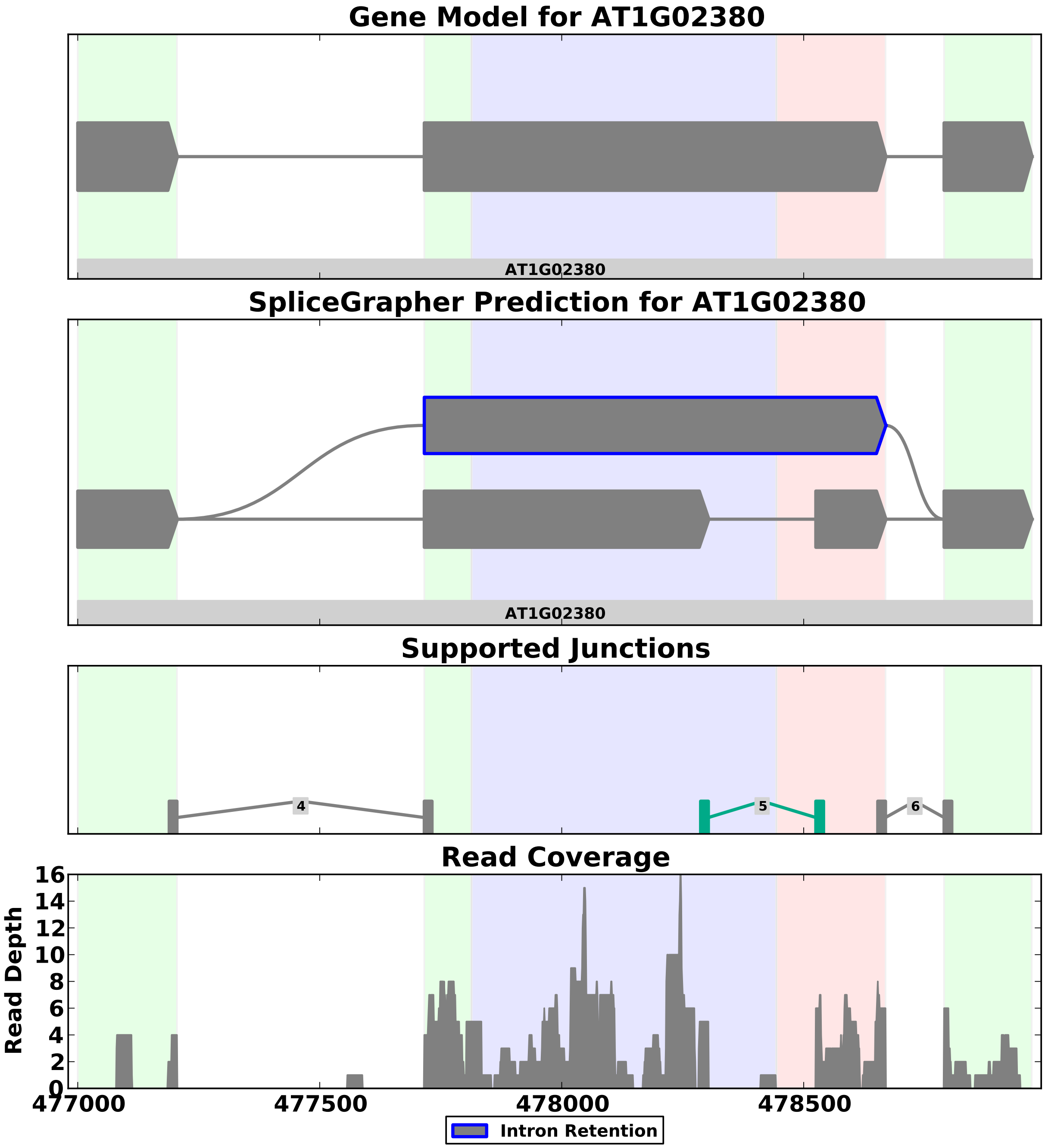

Supplementary Figure 11: *A. thaliana* gene AT1G02380 provides an example where the known splice form is the one with the intron retained (as opposed to the example shown in Figure 1, where the novel splice form has the intron as retained). This scenario of intron retention requires different evidence, namely the novel splice junction, shown in green in the figure. The boundaries of the exon from the gene model were used to infer the boundaries of the exons that flank the new intron.

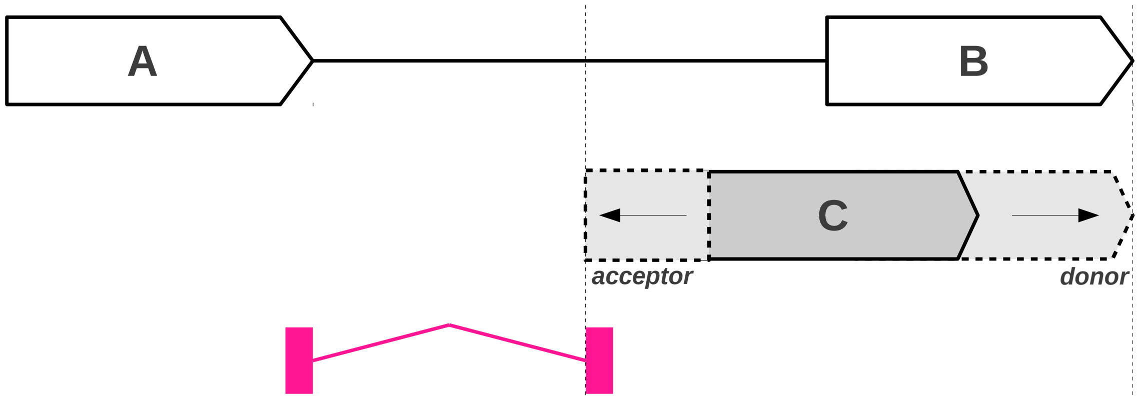

Supplementary Figure 12: The method SpliceGrapher uses to resolve an alternate acceptor site when a short-read exon (C) extends beyond another exon (B). If the exon C is not directly flanked by splice junctions (known or predicted from RNA-Seq data), SpliceGrapher will attempt to extend the exon to the nearest splice junctions, upstream and downstream. If such an extension is not supported by the data, the exon is considered unresolved.

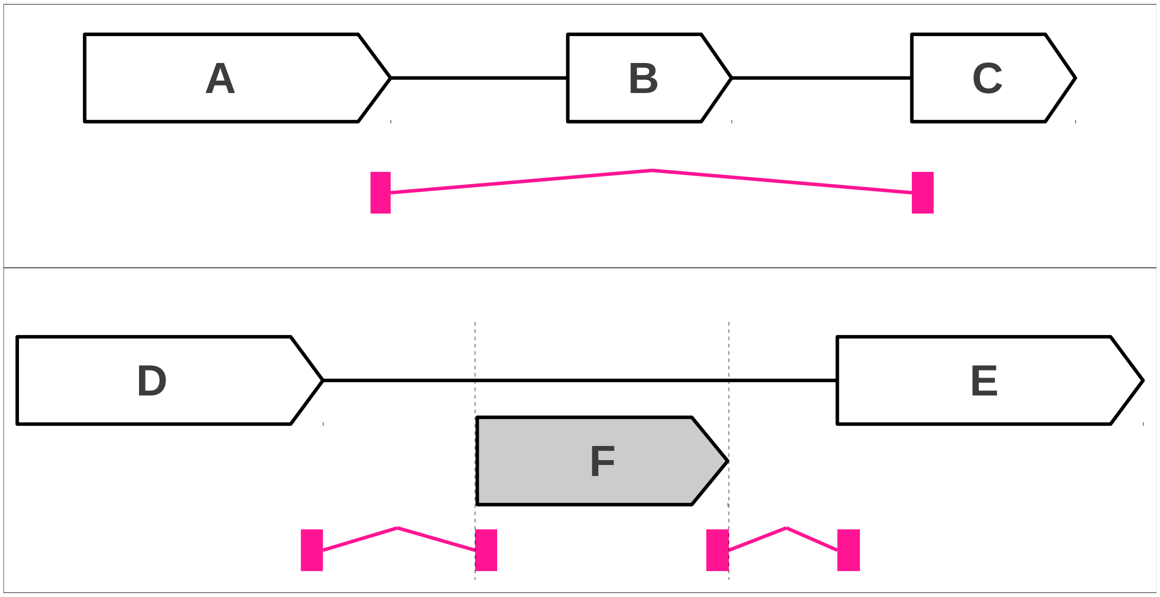

Supplementary Figure 13: Two scenarios in which SpliceGrapher will predict a skipped exon. In the top panel, a novel splice junction between exons A and C provides evidence that exon B is skipped in some transcripts. In the lower panel a novel exon, F, is contained within an intron in the graph. The new exon is flanked by novel splice junctions within the same intron, so SpliceGrapher uses the acceptor site from the upstream junction to resolve the exon's 5' boundary and the donor site from the downstream junction to resolve its 3' boundary. If SpliceGrapher is unable to resolve either boundary, the exon is unresolved.

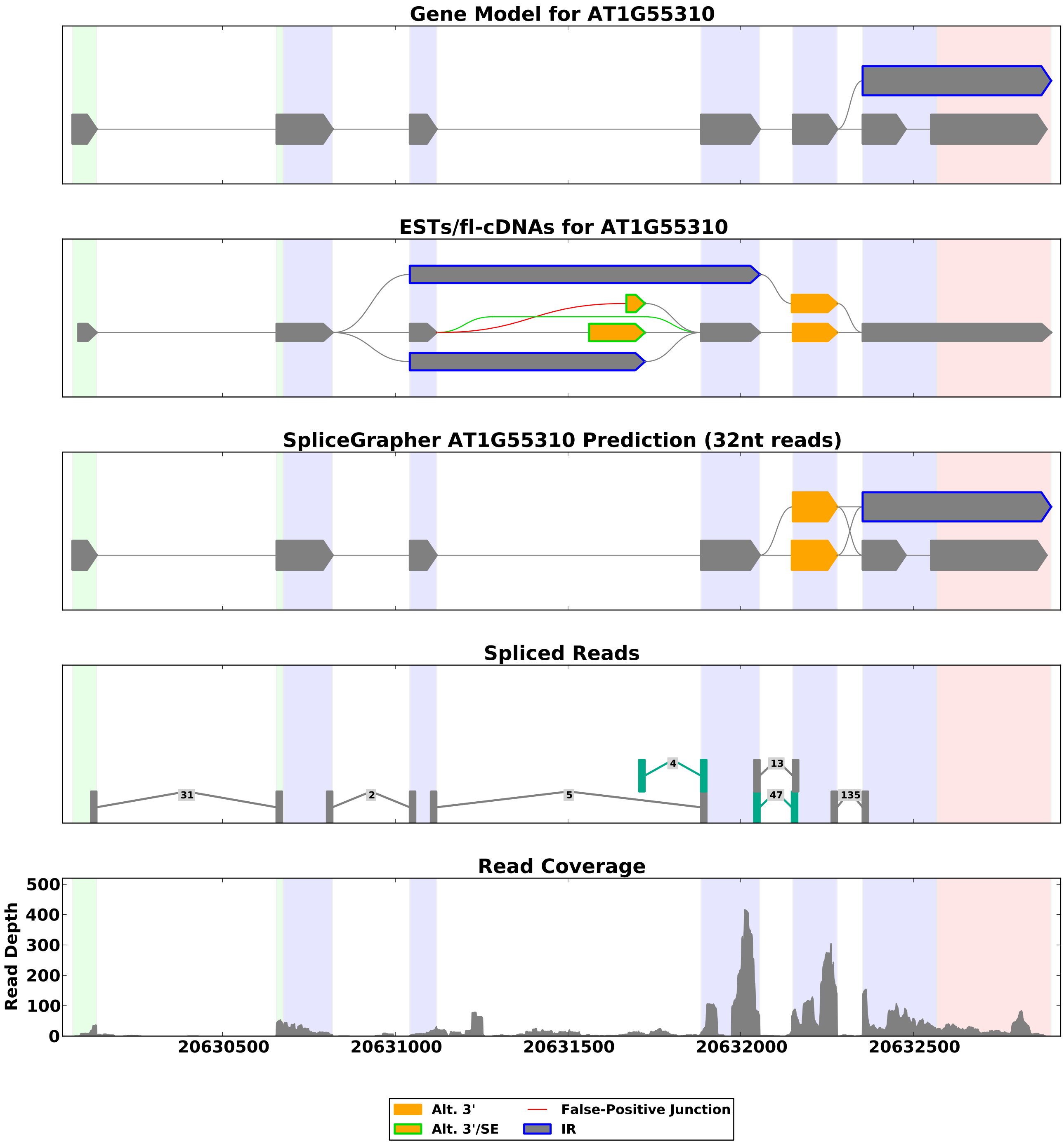

Supplementary Figure 14: Comparison between the gene models, EST/fl-cDNA alignments and SpliceGrapher's prediction the SR gene AT1G55310 in *A. thaliana*. From the RNA-Seq data, SpliceGrapher is able to predict one of the novel AS events in the ESTs. Note that SpliceGrapher identified a false positive splice junction in one of the EST alignments. It is a non-canonical AT site that is 2nt downstream of an AG dimer.

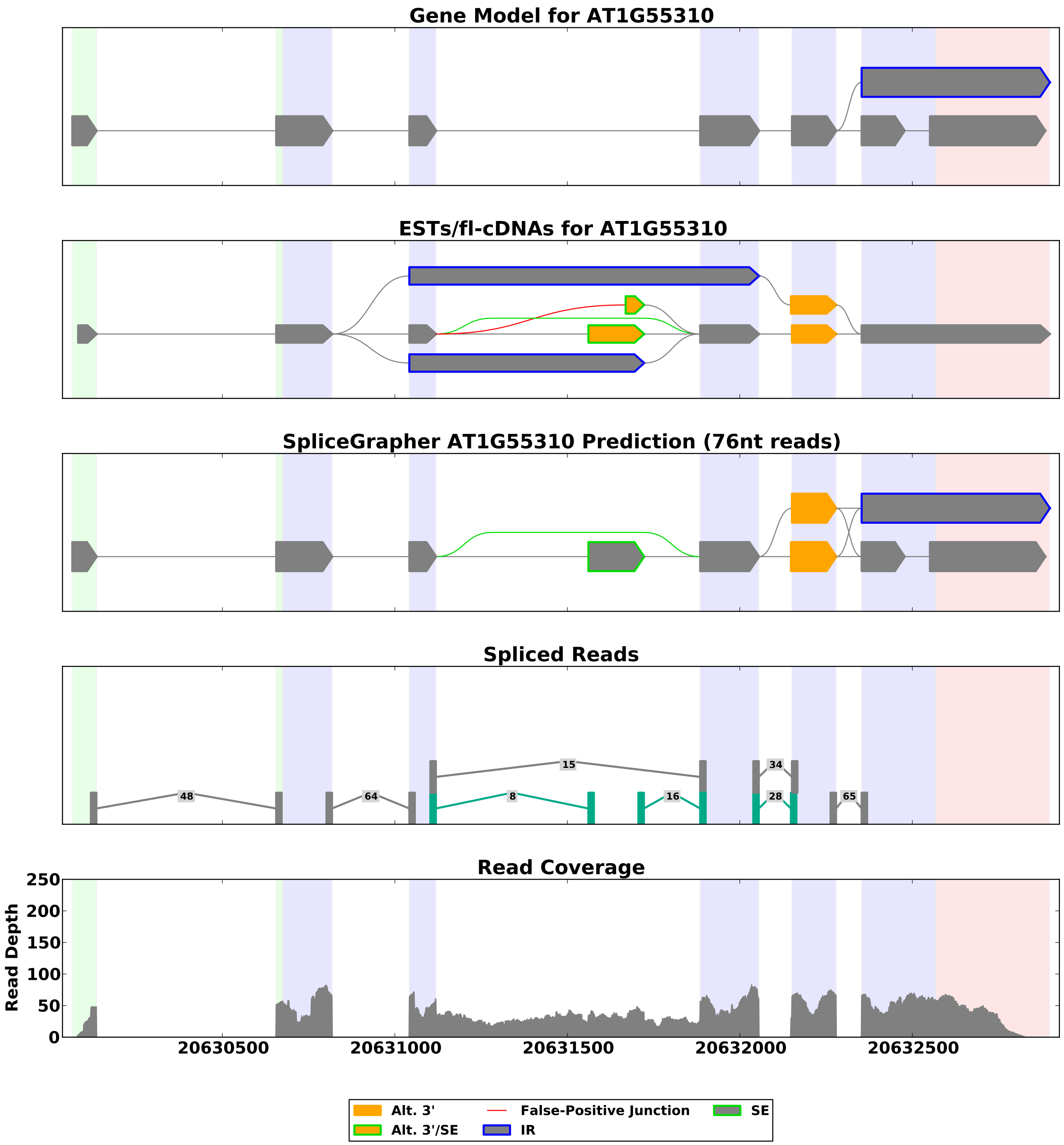

Supplementary Figure 15: Comparison between the gene models, EST/fl-cDNA alignments and SpliceGrapher's prediction for the SR gene AT1G55310 in *A. thaliana*. Here SpliceGrapher is able to predict more of the novel alternative splicing events found in the ESTs despite weaker read coverage than in the previous example. The longer reads (76nt) make it easier to identify unique locations for spliced reads. However, it is still not possible to identify which of three possible intron retention events are represented in the RNA-Seq data. (EST=expressed sequence tag; fl-cDNA=full-length complementary DNA, RNA-Seq=RNA sequences produced by next-generation sequencing)

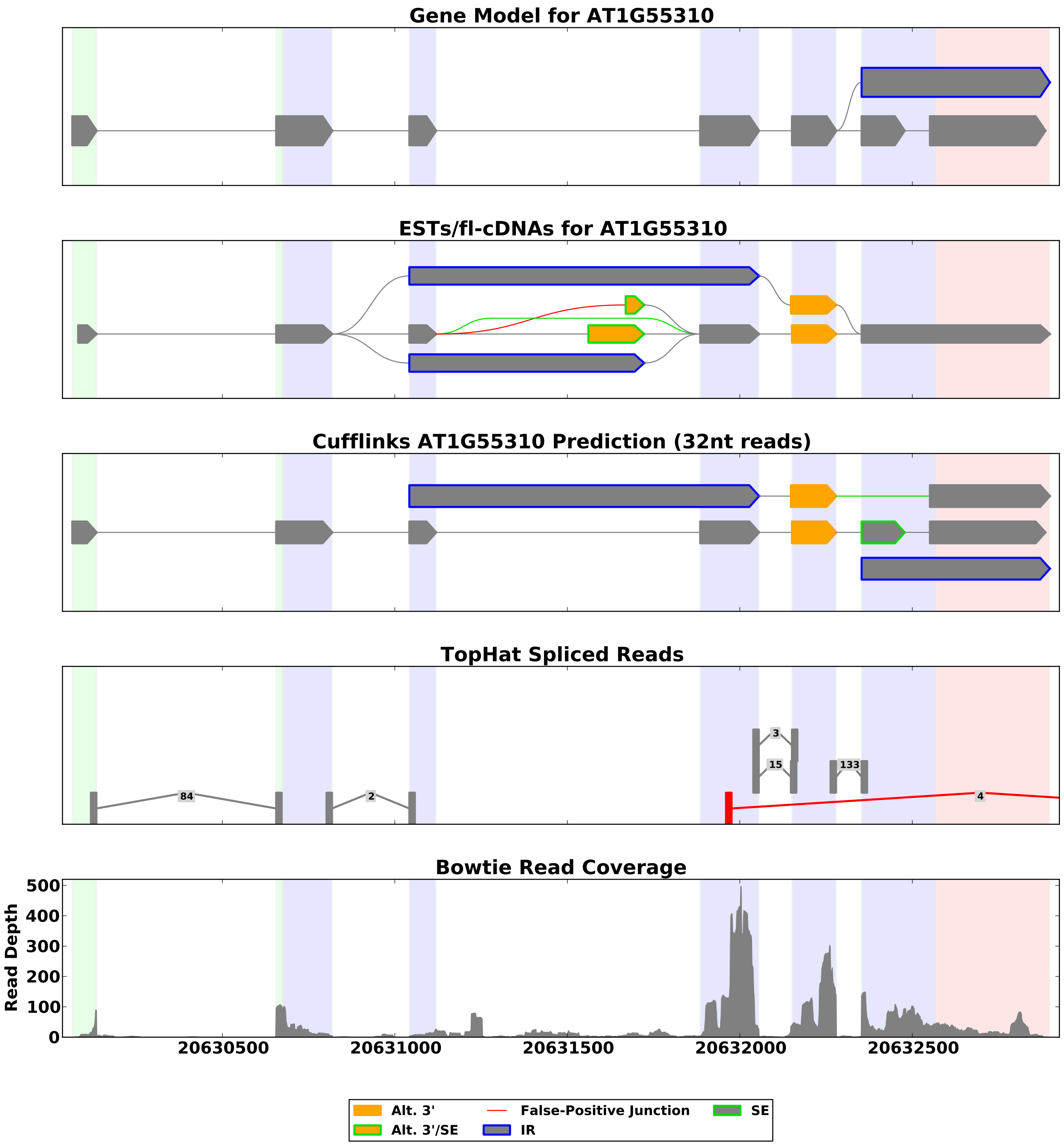

Supplementary Figure 16: Comparison between the gene models, EST/fl-cDNA alignments and the Cufflinks prediction for the SR gene AT1G55310 in *A. thaliana*. Given 32nt reads and gene models, Cufflinks predicts three fragments that include some of the alternative splicing from the gene models and two events found in the ESTs. It also predicts an exon skipping event that is not evident either in the ESTs or in the RNA-Seq data. (EST=expressed sequence tag; fl-cDNA=full-length complementary DNA; SR=serine-arginine, RNA-Seq=RNA sequences produced by next-generation sequencing)

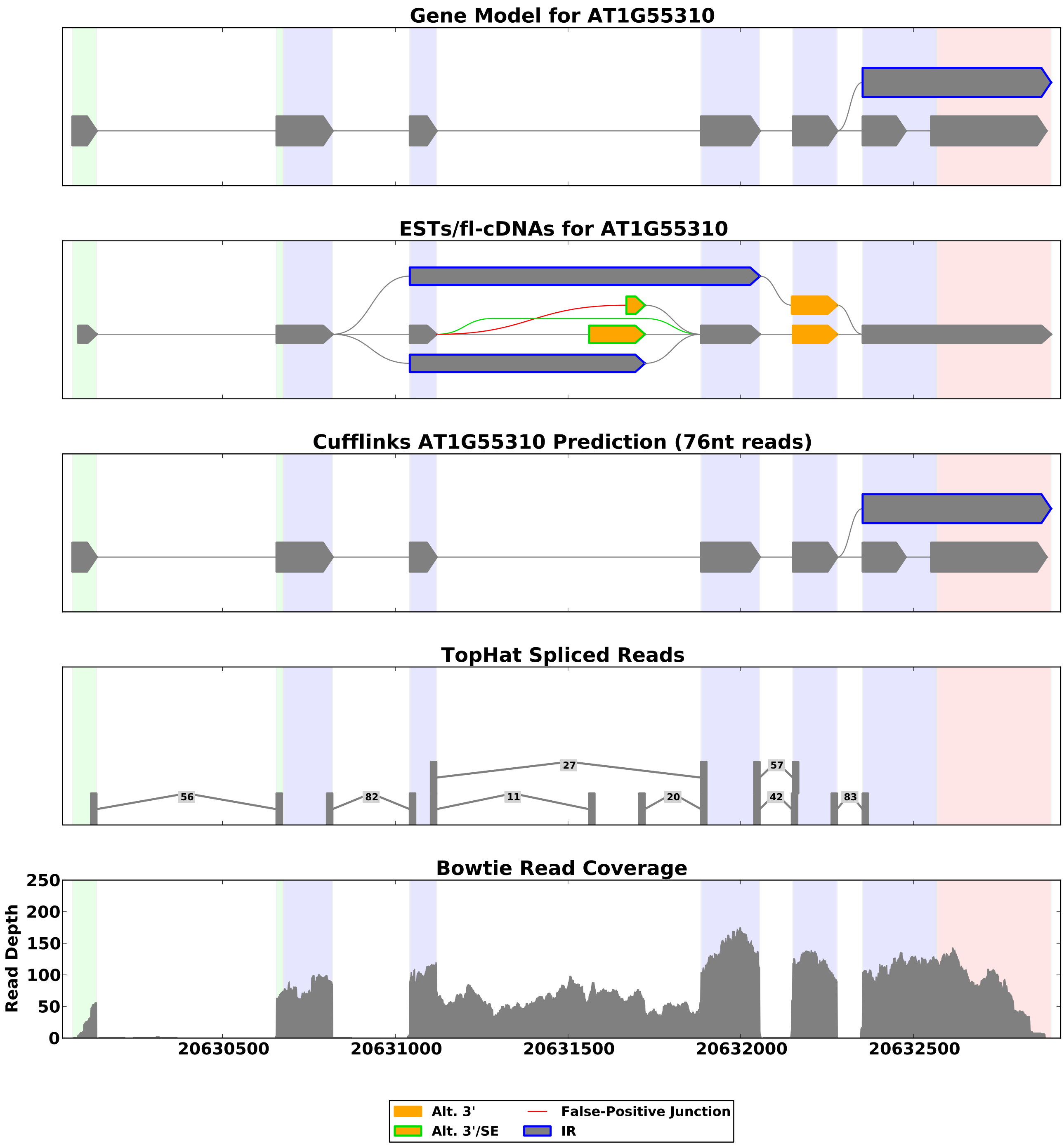

Supplementary Figure 17: Comparison between the gene models, EST/fl-cDNA alignments and the Cufflinks prediction for the SR gene AT1G55310 in *A. thaliana*. Given 76nt reads and gene models, Cufflinks predicts only the gene models and none of the events found in the ESTs. This is consistent with our other results which suggest that Cufflinks may not generalize well when provided gene model annotations. (EST=expressed sequence tag; fl-cDNA=full-length complementary DNA, SR=serine-arginine)
